# NPM1 phosphorylation-mediated telomere maintenance via stabilization of POLD3 in ALT-positive osteosarcoma: unraveling mechanisms and therapeutic opportunities

**DOI:** 10.7150/thno.108662

**Published:** 2026-01-22

**Authors:** Rui Zhao, Tingfang Li, Qiuhui Yang, Duo Jiang, Yanan Xue, Haomeng Kou, Qianqian Wang, Yuwen Wang, Xinyu Han, Wenbin Ma, Guowen Wang, Jinyan Feng, Xiuxin Han, Yancheng Liu, Yaqing Jing, Xin Geng, Fei Wang, Yang Liu, Qiang Zhang, Feng Wang

**Affiliations:** 1 Department of Genetics, School of Basic Medical Science, Tianjin Medical University, The Province and Ministry Co-sponsored Collaborative Innovation Center for Medical Epigenetics, Institute of Prosthodontics School and Hospital of Stomatology, Tianjin Medical University General Hospital, Tianjin, PR China.; 2 Tianjin Traditional Chinese Medicine Institute of Nephropathy, Tianjin Academy of Traditional Chinese Medicine, Tianjin, PR China.; 3 School of Inspection Ningxia Medical University Yinchuan, Ningxia, PR China.; 4 Department of Biochemistry, State Key Laboratory of Biocontrol, Sun Yat-sen University, Guangzhou, PR China.; 5 Department of Bone and Soft Tissue Tumors, Tianjin Medical University Cancer Institute and Hospital, National Clinical Research Center for Cancer, Tianjin, PR China.; 6 Department of Spinal Surgery, Tianjin Hospital, Tianjin, PR China.; 7 Department of Biochemistry and Molecular Biology, School of Basic Medical Science, Tianjin Medical University, Tianjin, PR China.; 8 Department of Neurology, Tianjin Medical University General Hospital, Tianjin, PR China.; 9 State Key Laboratory of Advanced Medical Materials and Devices, Tianjin Key Laboratory of Radiation Medicine and Molecular Nuclear Medicine, Tianjin Institutes of Health Science, Institute of Radiation Medicine, Chinese Academy of Medical Sciences & Peking Union Medical College, Tianjin, PR China.; 10 Department of Geriatrics, Tianjin Medical University General Hospital, Tianjin Geriatrics Institute, Tianjin, PR China.; 11 Tianjin Key Laboratory of Cellular and Molecular Immunology, Tianjin, PR China.

**Keywords:** NPM1, CST, ALT, telomere, BITR, POLD3

## Abstract

Maintaining telomere integrity is essential for cellular survival, and reactivation of telomerase or alternative lengthening of telomeres (ALT) represents a hallmark of cancer, ensuring replicative immortality. Osteosarcoma (OS), a malignancy in which many tumors rely on ALT for telomere maintenance, lacks effective therapeutic strategies targeting this pathway. This study aimed to identify and characterize novel molecular regulators of ALT activity and explore their potential as therapeutic targets in OS.

**Methods:** Immunohistochemistry was performed to evaluate the expression of phosphorylated NPM1 (pT199-NPM1) in OS tissues. Functional experiments including NPM1 knockdown and rescue assays were conducted to assess the impact of NPM1 on break-induced telomere replication (BITR) and cell viability in ALT-positive cells. Mechanistic studies involving phosphorylation analysis, ubiquitination assays, and co-immunoprecipitation were used to determine how ATR-mediated phosphorylation of NPM1 regulates POLD3 stability and its interaction with the CST complex. Pharmacological screening was performed to identify compounds that inhibit ALT activity, followed by *in vitro* proliferation assays and *in vivo* mouse xenograft experiments to evaluate therapeutic efficacy and synergy with doxorubicin.

**Results:** We identified pT199-NPM1 as a novel, highly expressed protein factor in ALT-positive OS tissues. NPM1 depletion impaired break-induced telomere replication and significantly reduced the viability of ALT-positive cells. ATR signaling phosphorylated NPM1 at Thr199, which stabilized POLD3 by preventing its ubiquitin-mediated degradation. Recruitment and function of pT199-NPM1 at telomeric damage sites required STN1, defining a CST/pT199-NPM1/POLD3 regulatory axis essential for ALT activity. Clinically, elevated Thr199 phosphorylation correlated with poor survival in OS patients, while expression of a phosphorylation-deficient T199A mutant failed to sustain ALT telomere maintenance. Pharmacological screening identified EPZ-6438, an EZH2 inhibitor, as a potent ALT suppressor that reduced NPM1 transcription, inhibited homologous recombination-mediated telomere synthesis, and suppressed OS cell proliferation. In mouse xenografts, EPZ-6438 enhanced OS cell sensitivity to doxorubicin, suggesting therapeutic synergy.

**Conclusions:** This study uncovers a novel CST/pT199-NPM1/POLD3 regulatory module that is critical for ALT telomere maintenance in OS. Targeting NPM1 or its downstream effectors effectively suppresses ALT activity and enhances chemotherapy response. These findings provide new mechanistic insights into telomere regulation in ALT-positive tumors and highlight the therapeutic potential of NPM1-centered pathways in OS.

## Introduction

Telomeres are protective caps located at the ends of eukaryotic linear chromosomes. During each round of cellular division, they undergo shortening due to the end replication problem, which eventually leads to permanent growth arrest, known as senescence 1-3. To achieve proliferative immortality, most cancer cells add back telomeric DNA *de novo* using telomerase 4. However, the remaining 10-15% of tumor cells, including some with a particularly aggressive nature and poor prognosis, use a recombination-based telomere maintenance mechanism called alternative lengthening of telomeres (ALT) 5, 6. ALT cells are characterized by abnormally heterogeneous telomeres, clustering of ALT-associated PML bodies (APBs), and accumulation of extrachromosomal circular DNA of telomeric repeats (C-circles) 7-10.

Recent breakthroughs in telomere biology have provided insights into the key factors involved in ALT. Chromatin remodeling protein ATRX, histone chaperone DAXX 11, 12, shelterin components TRF1/TRF2 13-15, and various protein factors involved in damage repair and DNA replication, including BRCA1, the MRN (MRE11-RAD50-NBS) complex, RAD52, SLX4, WRN and BLM, have been reported to contribute to ALT telomere maintenance 16-19. ALT telomeres exhibit higher levels of spontaneous double-strand breaks (DSBs) and telomere replication stress compared to telomerase-positive cancer cells 20, 21. This suggests that ALT may be initiated by replication stress or DNA damage at telomeres, leading to a complex process known as break-induced telomere replication (BITR). BITR involves the conservative replication of telomeric DNA 21. In response to ALT-associated DSBs, a replication machinery comprising replication factor C (RFC), proliferating cell nuclear antigen (PCNA), and polymerase delta subunit 3 (POLD3) is assembled. This RFC-PCNA-POLD3 axis facilitates break-induced telomere synthesis, with POLD3 functioning as a key effector of telomeric DNA extension 22. While several crucial events that activate ALT have been identified, further research is required to determine how BITR is regulated at telomeres.

Recent studies have revealed the potential role of the CST complex in ALT telomere maintenance 23. The CST complex, composed of CTC1, STN1, and TEN1 proteins, plays a critical role in regulating telomere homeostasis and replication in mammalian cells. Specifically, the CST complex has been found to be localized at DNA damage sites in a 53BP1-and shieldin-dependent manner during telomeric DNA breaks, indicating its importance in damaged telomeric DNA repair 24. Moreover, CST was found to be located at APBs in ALT cells and to form a functional complex. Depletion of any CST component can diminish the formation of C-circles 23, further supporting its involvement in the ALT pathway. Nevertheless, more research is needed to fully understand the connection between CST and ALT.

To further explore the role of CST in ALT telomere synthesis, a high throughput Bimolecular Fluorescence Complementation (BiFC) screening assay is used in our current study to identify its co-factors in living ALT cells. We observed that a nucleolar phosphoprotein NPM1 preferentially interacted with CST in ALT cells compared to telomerase-positive cells. Previous studies have reported that NPM1 shuttles between the nucleolus and nucleoplasm and is vital for multiple cellular functions, including ribosome synthesis, centrosome duplication, cell proliferation, apoptosis, and DNA repair 25-29. It is overexpressed in a variety of solid tumors 30-33, whereas its deficiency impairs DNA repair response and enhances the tumor radiosensitization 34. Previous studies have shown that NPM1 is a DNA damage induced chromatin binding protein that promotes p53 stabilization by competitively binding to the p53 E3 ligase MDM2 35-38. Moreover, it was also observed to interact with BRCA1 to participate in the homologous recombination repair process 39. However, there is currently no direct evidence that NPM1 is involved in telomere damage repair. The detailed mechanism of how NPM1 participates in telomeric DNA damage repair and ALT still needs further investigation.

Here, this study employed BiFC technology to identify CST co-factors involved in recombination-based telomere synthesis in ALT cells. Surprisingly, NPM1 was identified as a CST binding partner that is essential for telomere maintenance in both ALT and telomerase-positive cells after telomere breaks. In contrast to the dispersed distribution of NPM1 in the nucleolus, phosphorylated Thr199-NPM1 is recruited to damaged telomeres, possibly via its interaction with CST. The phosphorylation of Thr199 and its colocalization with telomeres could be abolished by ATR inhibition. Additionally, high expression of phosphorylated NPM1 was observed to be closely associated with reduced overall survival in patients with ALT-positive OS. The study also demonstrated that phosphorylated NPM1 at Thr199 stabilizes the recruitment of its binding partner POLD3 to telomeres. In order to broaden the clinical applications of this theory, a high-throughput screening system was used to identify compounds that inhibit NPM1 transcription and tazemetostat (EPZ-6438) was selected, which exhibited the most significant inhibition of NPM1 at the transcriptional level. Treatment with EPZ-6438 significantly repressed the ALT activity, in turn, inhibited OS tumor cells proliferation and sensitized Dox treatment in mouse xenograft model. Overall, these findings reveal a previously undiscovered mechanism of NPM1 in ALT and suggest that targeting NPM1 may be a promising strategy for cancer therapy.

## Material and Methods

### Cell culture and compounds

U2OS and HEK 293T cells were cultured in DMEM (Corning, USA), HeLa and SaoS2 cells were cultured in RPMI 1640 (Corning) and McCoy's 5A medium (HyClone, USA), respectively. U2OS/MTX300 cells were cultured in DMEM (Corning) containing 300 ng/mL Methotrexate (MTX). All of them supplemented with 10% fetal bovine serum (Gibco, USA) and 1% penicillin/streptomycin (HyClone, USA). MTX (M8917) was purchased from Solarbio (China). Proteasome inhibitor MG132 (MB5137) were purchased from Meilunbio. 5 or 10 μM MG132 was used in U2OS cells for 24 h. Inhibitor of ATM (KU55933) and ATR (VE-821), doxorubicin (S1208) and EPZ-6438 (S7128) were purchased from Selleck Chemical (USA). HeLa cells were treated with 10 μM ATMi or ATRi for the experiments for 24 h. 0.075 μM doxorubicin was used in U2OS cells for 72 h. 10 or 100 μM EPZ-6438 was used in U2OS or HeLa cells for 2 or 5 days. Cells were infected with lentiviruses expressing *NPM1*-targeting shRNA (5'-GCGCCAGTGAAGAAATCTATA-3' and 5'-CCTAGTTCTGTAGAAGACATT-3') and Scramble shRNA (5'-CCTAAGGTTAAGTCGCCCTCG-3') and selected with puromycin (Solarbio). They were attached into pLKO.1 vector. Full length human *NPM1*, *STN1*, *POLD3* cDNA was subcloned into pcDNA3 HA or pCDH Flag, pcDNA3 × Flag and plenti 3 × Flag vector, respectively. Mutants were generated by site-directed mutagenesis and subcloned into pcDNA3 HA and MSCV-IRES-Thy1.1 vector. The underlined mutations (ACGAGATGCTCCAGCCAAAAA) were introduced in the* NPM1* coding region. Cas Tel plasmid, which consists of the Cas9 enzyme and sgRNA-targeting telomeric sequences (5′-CACCGTTACCGTTAGGGTTAGGGTTA-3′) was a gift from professor Yong Zhao (Sun Yat-sen University, Guangzhou).

### Bimolecular fluorescence complementation (BiFC) assay

BiFC assay was performed in the base of previous described 40, 41. The target gene *STN1* was fused to a retroviral expression vector, pBabe-CMV-DEST-YFPn-neo, which tagged with N-terminal of *YFP* (yellow fluorescent protein, 1-155 aa) to form *STN1*-*YFP*n and 18,000 open-reading frames (ORFs) from the hORFeome v7.1 library tagged with C-terminal of* YFP* (156-239 aa, *YFP*c) with a mixture of pBabe-CMV-YFPc-DEST-puro. Then the virus of *STN1*-*YFP*n and *YFP*c-prey packaged in HEK 293T cells with 10 μg of *STN1*-*YFP*n plasmid/*YFP*c plasmids, 7.5 μg of pSPAX2 and 2.5 μg of pMD2G packaging plasmid were diluted with Opti-MEM (Gibco) for 20 min. After transfection of 8 h, the culture medium was replaced by fresh medium and virus was collected after 48 h. Here, we obtained a database of viruses of *YFP*-c plasmids. We transferred *YFP*c to U2OS and HeLa cells expressing *STN1*-*YFP*n. We used empty vector pBabe-CMV-YFPc-DEST-puro and pBabe-CMV-DEST-YFPn-puro as a negative control, which cannot interact with each other and produce complementary fluorescence. Protein expression vectors pBabe-CMV-YFPc-POT1-puro and pBabe-CMV-TPP1-YFPc-puro were used as positive controls because POT1 and TPP1 have been shown to interact with each other. Flow cytometry was used to screen positive cells for three rounds. Then RNA was extracted and reverse transcribed to cDNA. We used specific primers of N-terminal and C-terminal sequences of pBabe-CMV-puro to obtain PCR products.

### Silver staining and mass spectrometry

HEK 293T cells were transiently transfected with Flag-*NPM1* using polyethyleneimine (PEI) (Polysciences, USA) in the Opti-MEM medium (Gibco). Cells were lysed in 1% NP-40 buffer (50 mM Tris-HCl, pH 8.0, 150 mM NaCl, 1 mM PMSF) and then incubated with anti-Flag M2 affinity gel beads (Sigma-Aldrich, USA) overnight. Next, the beads were washed with 1% NP-40 buffer six times. Then 0.2 mg/mL Flag peptide (Sigma-Aldrich) was added to the beads. The eluted protein samples were separated by SDS-PAGE followed by silver staining manufacturer's protocol (Thermo, USA). The specific bands were cut to perform mass spectrometry.

### Purified proteins and GST pull-down assay

GST-NPM1 and fragments were cloned in pGEX-6P-1, His-STN1 and His-TEN1 were cloned in pET28a. GST-NPM1 and fragments, His-TEN1 were expressed by isopropyl-L-thio-B-D-galactopyranoside (IPTG) (Solarbio, China) induction at 16 ℃ for 12 h followed by expressing of His-STN1 at 22 ℃ for 12 h. Flag-CTC1 were cloned in pFastBac and it is obtained by baculovirus expression system. For pull down assay, purified GST-NPM1 and fragments were mixed with His-STN1, His-TEN1, Flag-CTC1 and glutathione agarose beads in PBS buffer. After rotation at 4 ℃ for 2 h, the beads were washed for six times, boiled in SDS loading buffer with 0.1 mol/L DTT, and resolved by SDS-PAGE separation followed by Western blotting.

### Cell counting kit-8 (CCK-8) assay

Cell viability was measured with CCK-8 (Biosharp). In briefly, U2OS, HeLa and shNPM1 clones were seeded in 96-well plates (1 × 10^3^/well) and incubated at 37 ℃. The medium was replaced with fresh medium containing 10% CCK-8 on different days and the 450 nm wavelength absorbance was measured with microplate reader (BIO-RAD).

### EdU labeling of telomere DNA synthesis

U2OS cells were synchronized in the G2/M phase with 2 mM thymidine (Sigma, USA) for 21 h. The next day, cells were washed three times with 1×PBS and medium was replaced with fresh DMEM for 8 h. Then cells were treated with 10 μM RO-3306 (MedChemExpress) for 18 h and pulsed with 10 μM EdU (Trans, China) for 2 h before cells harvest. Cell cycle of a subset of cells were analyzed by flow cytometry. Coverslips were washed with 1 × PBS, then fixed with 3.7% paraformaldehyde for 15 min. After permeabilization (0.5% Triton X-100 for 20 min), cells were washed with 3% BSA in 1 × PBS twice and incubated with EdU reaction buffer (Trans) for 30 min at room temperature. Coverslips were then washed with 1 × PBS and incubated with Alexa 488 conjugated (CCCTAA)_4_ PNA probe.

### Cell synchronization and treatment

Cell synchronization for MiDAS analysis was performed with modifications to previously described methods 42, 43. To ensure sufficient expression or drug exposure during synchronization, cells were first transfected with either empty vector or NPM1 expression plasmids, and/or treated with EPZ-6438 for a total duration of 48 h prior to sample collection. For synchronization, cells were treated with or without low-dose (0.2 μM) aphidicolin (APH, Sigma-Aldrich) for 16 h. To arrest cells in late G2 phase, the CDK1 inhibitor RO-3306 (9 μM) was added either simultaneously with APH or during the last 8 h of APH treatment. After synchronization, cells were released from the G2 block by washing three times with room-temperature PBS and incubated in fresh pre-warmed medium (37 ℃). EdU (10 μM) was included in the release medium to label ongoing DNA synthesis during the release period (30 min). Mitotic cells were collected by mitotic shake-off and harvested by centrifugation for metaphase chromosome spread preparation and subsequent EdU detection and FISH analysis.

### Co-Immunoprecipitations (Co-IP)

HEK 293T cells were transiently transfected with HA-tagged wild-type (WT) or T199A NPM1 in the presence or absence of Flag-tagged STN1 or POLD3. Cells were harvested 48 h later and lysed in 1% NP-40 buffer on ice for 30 min and then centrifuged at 4 ℃ for 10 min. A total of 80 µL of cleared lysate was removed and denatured for 10 min to serve as the input sample, while the remaining lysate was incubated with 30 µL of anti-Flag agarose beads (Sigma, USA) overnight at 4 ℃ Then agarose beads were washed six times with 1% NP-40 buffer and denaturated for 10 min at 100 ℃ with 2 × SDS-PAGE loading buffer for SDS-PAGE analysis.

### siRNA transfection

siRNA oligonucleotides targeting *NPM1* (5'-AGGUGGUUCUCUUCCCAAATT-3'), *STN1* (5'-GAUCCUGUGUUUCUAGCCU-3'), and non-targeting control (5'-UUCUCCGAACGUGUCACGUTT-3') were purchased from GenePharma. 2 × 10^5^ cells were seeded per well of 6-well plate. 2.5 μL RNA duplexes (final concentration 50 nmol/L) were transfected into cells by using 5 μL Lipofectamine RNAiMax (Invitrogen, USA) per well. siRNAs and RNAiMAX were diluted in Opti-MEM (Gibco, USA) and added to culture-containing cells. Cells were harvested 72 h after transfection.

### Western blotting

Cells were harvested with trypsin and washed in 1 × PBS. Cells lysed in RIPA buffer supplemented with protease inhibitors (MedChemExpress) on the ice for 30 min and then centrifuged at 4 ℃ for 20 min. Cell lysates were denaturated for 10 min at 100 ℃, separated by SDS-PAGE electrophoresis and transferred to PVDF membrane. Primary antibody incubated overnight at 4 ℃. For secondary antibodies, HRP-conjugated anti-rabbit or mouse (Immunoway) was used for 1 h at room temperature. Immunoreactivity was detected by chemiluminescence.

### Antibodies

Antibodies used in this study were as follows: anti-NPM1 (YT3208 Immunoway, 1:1000 and Cat No. 60096-1-lg proteintech, 1:500), anti-STN1 (ab252855 Abcam, 1:500), anti-γ-H2AX (05-636 Milipore, 1:1000), anti-53BP1 (ab175933 Abcam, 1:1000), anti-ATM (2873S Cell Signaling Technology, 1:1000), anti-ATR (2790S Cell Signaling Technology, 1:1000), anti-Chk1 (ab47574 Abcam, 1:500), anti-p-ATM (5883S Cell Signaling Technology, 1:1000), anti-p-ATR (2853S Cell Signaling Technology, 1:1000), anti-p-Chk1 (12302S Cell Signaling Technology, 1:1000), anti-pT199-NPM1 (3541S Cell Signaling Technology, 1:1000), anti-POLD3 (21935-1-AP Proteintech 1:1000), anti-RAD52 (sc-365341 Santa Cruz, 1:100), anti-GAPDH (AF0911 Affinity, 1:5000), anti-β-tubulin (AC008 Abclonal, 1:2000), anti-HA-tag (3724S Cell Signaling Technology, 1:1000), anti-Flag-tag (2368S Cell Signaling Technology, 1:500), anti-GST-tag (10000-0-AP Proteintech, 1:1000), anti-His-tag (AH367 Beyotime, 1:1000), anti-PML (sc-5621 Santa Cruz, 1:500), Alexa Fluor 555 goat anti-rabbit (A21428 Lifetechnologies, 1:2000), Alexa Fluor 555 goat anti-mouse (A21422 Lifetechnologies, 1:2000), Alexa Fluor 488 goat anti-rabbit (A11008 Lifetechnologies, 1:2000), Alexa Fluor 4488 goat anti-mouse (A11001 Lifetechnologies, 1:2000).

### Immunofluorescence (IF)

Cells on glass coverslips were washed once in 1 × PBS and fixed with 2% PFA for 20 min. Fixed cells were permeabilized with 0.1% Triton X-100 for 20 min and then incubated in blocking solution (0.5% BSA in 1 × PBS) for 1 h. Then it was incubated in blocking solution containing primary antibodies for 1 h, washed four times with 0.1% Tween-20 in 1 × PBS (PBST) for 5 min each and incubated with Alexa coupled secondary antibodies (Invitrogen) diluted in blocking solution for 1 h at room temperature. Then, cells were washed four times with PBST for 5 min each again and blocked with DAPI.

### Immunofluorescence with fluorescent *in situ* hybridization (IF-FISH)

After permeabilized with 0.1% Triton X-100, cells were washed twice with 1 × PBS for 5 min each and coverslips dehydrated with washes in 70%, 90% and 100% EtOH for 2 min. Next, 30 μL per coverslip of hybridization mix containing Alexa 488 conjugated (CCCTAA)_4_ PNA probe were used. DNA was denatured at 80 ℃ for 3 min and incubated for 2 h at room temperature. Then cells were washed twice with Wash Solution I (70% deionized formamide, 0.1 M Tris-HCl pH 7.2 and 1 × blocking regent) for 15 min and three times with 0.1% TBST for 5 min each and incubated in blocking solution for 1 h. Next, as above described, cells were incubated with primary and secondary antibody and blocked with DAPI.

### Quantitative fluorescence *in situ* hybridization (Q-FISH)

After treating U2OS cells with colchicine (0.1 mg/mL) for 6 h, mitotic cells were harvested. FISH was performed on methanol/acetic acid-fixed (3:1) metaphase cells using the TelC-Cy3 probe (Panagene, Dacjeon, Korea). Fluorescence from chromosomes and telomeres was digitally imaged on an Olympus inverted microscope, with telomere length represented by telomere fluorescence intensity and analyzed using the TFL-TELO program.

### Flow cytometry

U2OS cells were transiently transfected with Thy1.1-WT and T199A *NPM1*. After 48 h, cells were collected for flow cytometry analysis of Thy1.1 expression. Briefly, cells were harvested and washed with 1 × PBS and then resuspended in 1 × PBS. Next, cells were incubated with anti-mouse Thy1.1 PerCP-Cyanine5.5 (Invitrogen) for 30 min away from light and washed twice with 1 × PBS. Cells were fixed with 4% PFA, and green fluorescence was analyzed by flow cytometry.

### C-circle assay

C-circle assay was slightly modified in the base of previous described 44. DNA was diluted in double distilled water and concentrations were exhaustively measured to the indicated quantity (30 ng of ALT cells, 60 ng of ALT-negative cells) using a Nanodrop (ThermoFisher, USA). Samples were combined with 1 × Φ29 buffer (NEB), BSA, 1 mM dNTP mix (Vazyme). Samples were incubated at 30 °C for 8 h and followed by heat-inactivation at 65 ℃ for 20 min. Amplification products were dot-blotted onto 2 × SSC-soaked nylon membranes (GE Healthcare) and hybridized with a DIG-labeled probe (CCCTAA)_4_ for 10 h. Blots were detected by Tanon 5200 and quantified using ImageJ software.

### Immunohistochemistry (IHC)

Normal bone tissue microarray (LN020Bn01), OS tissue microarray (L1024901) (Bioaitech, China) and bone tissues from OS patients were deparaffinized followed by antigen retrieval. The primary antibody against pT199-NPM1was diluted 1:200, and these sections were incubated with it at 4 ℃ overnight. After washing with 1 × PBS for 3 times, slides were treated with HRP-linked secondary antibody for 1 h at room temperature. IHC images were quantified by Image-Pro Plus 6.0 software.

### RNA extraction and quantitative real-time PCR (qRT-PCR)

The RNA of the cells was extracted according to the instructions of Promega (USA) RNA extraction kit. Next, according to the manufacturer's protocol, RNA samples were reverse transcribed into cDNA using ABScript III Reverse Transcriptase (ABclonal, China). qRT-PCR analysis was performed using the 2×Universal SYBR Green Fast qPCR Mix (ABclonal, China) and glyceraldehyde-3-phosphate dehydrogenase (*GAPDH*) was used for normalization. All primer were synthesized by Sangon Biotech (Shanghai, China) Co., Ltd and the sequences are *NPM1-*F: 5'-GGAGGTGGTAGCAAGGTTCC-3', *NPM1*-R: 5'-TTCACTGGCGCTTTTTCTTCA-3', *BRCA1*-F: 5'-ACAGCTGTGTGGTGCTTCTGTG-3', *BRCA1*-R: 5'-CATTGTCCTCTGTCCAGGCATC-3', *BRCA2*-F: 5'-TTCCCTCTGCGTGTTCTCACAA-3', *BRCA2*-R: 5'-GCCATCCACCATCAGCCAACT-3', *RAD51*-F: 5'-GCATGAAGTACTGGCGTGAACA-3', *RAD51*-R: 5'-ACTCGGCCTCTAATCAGTCTGG-3', *GAPDH*-F: 5'-GCCACATCGCTCAGACAC-3', *GAPDH*-R: 5'-GCCCAATACGACCAAATCC-3'.

### Screening strategy of NPM1 inhibitors based on the fluorescence intensity of GFP-tagged NPM1 in U2OS cells

Plasmids containing the upstream 1000 bp promoter sequences of NPM1 and GAPDH fused with GFP separately were transfected into U2OS cells. High-throughput screening was performed using the Perkin Elmer G3 system. Briefly, cells were seeded in 96-well plates at a density of 8000 cells per well using an automatic liquid handler integrated into the system. The plates were then incubated in a 37 °C incubator for 12 h. On the second day, compounds from the L1200-Epigenetics Compound Library (L1200, TOPSCIENCE) and the Bioactive Compound Library (TM003, Tianjin Medical University) were added to the 96-well plates at final concentrations ranging from 1-50 μM. Cells treated with candidate drugs were cultured for an additional 24 h. The NPM1-fused-GFP fluorescence intensity was then recorded and normalized relative to the intensity of GFP in GAPDH-fused-GFP cells using the Opera Phenix Plus High-content Screening System (Perkin Elmer, HH14001000).

### Xenografts

Female BALB/c nude mice (5 weeks old) were subcutaneously injected with 2 × 10^6^ U2OS/MTX300 or sh*NPM1* cells in 100 μL PBS containing 50% Matrigel (Corning). Nude mice were randomly divided into eight groups and treated with 150 μL vehicle control, doxorubicin (7.5 mg/kg; intravenous), EPZ-6438 (200 mg/kg; oral gavage), or in combination. EPZ-6438 were given twice a day for 25 days. Once the average volume of tumors reached 100 mm^3^, doxorubicin was given intravenously once every 2 days for 2 weeks. Tumor volumes were measured every 2 days with calipers and calculated with the formula, *V*= *1/2*(length × width^2^). After termination of treatment, mice were euthanized, tumors were resected and weighed.

## Results

### NPM1 interacts with the CST complex

It has been reported that the mammalian CST (CTC1-STN1-TEN1) complex plays an essential role in maintaining telomeres and is involved in various telomere damage repair processes 45, 46. Moreover, CST has been identified to be significantly localized to telomeres in cells that maintain their telomeres via ALT 23. Here, to determine how CST impacts ALT telomere replication and whether additional co-factors are required, we conducted a BiFC assay to systematically identify proteins interacting with CST in ALT cells compared to telomerase-positive cells.

**Figure 1:**
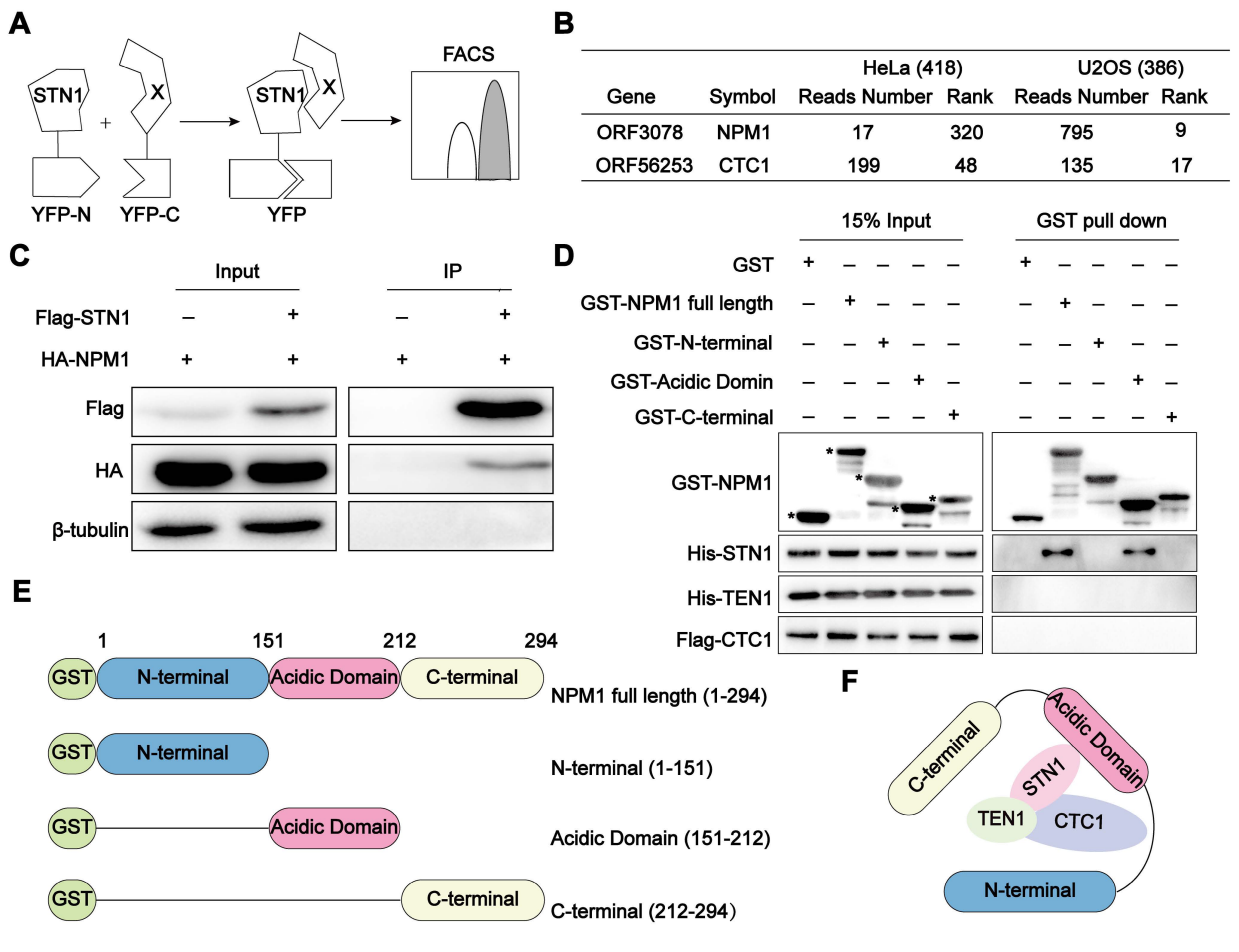
** NPM1 interacts with the CST complex. (A)** Schematic diagram of the Bimolecular Fluorescence Complementation (BiFC) assay.* STN1* and 18000 human genes (X) were subcloned into YFPn (N-terminal of yellow fluorescent protein) and YFPc (C-terminal of yellow fluorescent protein), respectively. When STN1 interacts with X, fluorescence complementation can be detected by flow cytometry.** (B)** BiFC screening showing the proteins that interact with STN1. The table displays the number of reads and rank of NPM1 and CTC1, which are proportional to the intensity of the protein interaction. The greater the number of reads, the lower the rank is displayed. CTC1 serves as a positive control.** (C)** The immunoprecipitation and Western blotting analysis confirming the interaction between HA-NPM1 and Flag-CST (STN1). β-tubulin serves as a loading control. n = 3 biological replicates. **(D)** GST-pull down assay showing the direct interaction between GST-NPM1 and 6×His-STN1. GST-NPM1 was immobilized on beads to capture the indicated proteins, including 6×His-STN1, 6×His-TEN1, and Flag-CTC1. GST serves as a negative control. Asterisks (*) denote the purified GST-NPM1 domain fragments. n = 3 biological replicates.** (E)** Schematic diagram of the full-length or truncated NPM1 proteins used for the pull-down assays. **(F)** A model illustrating the interaction between NPM1 and CST.

The advantage of BiFC lies in its ability to authentically and visually capture the transient and weak interactions between proteins. Cells (U2OS or HeLa) co-expressing STN1-YFPn protein and the human cDNA library (hORFeomeV7.1) encoded YFPc were detected by flow cytometry (Figure 1A) as described 40. The YFP-positive cells were collected and cultured after three rounds of sorting. The tagged cDNA was subsequently sequenced, and we identified 386 candidate genes in U2OS cells and 418 genes in HeLa cells, the encoding proteins of which have the potential to interact with STN1, using the number of reads as references. Notably, among the identified STN1-associated proteins, NPM1 emerged as a top-ranking candidate specifically in ALT-positive U2OS cells. While its interaction with STN1 ranked relatively low in telomerase-positive HeLa cells (320th), it rose sharply to 9th in U2OS cells, indicating a pronounced ALT-specific enrichment. As an internal validation, STN1 also interacted with CTC1, consistent with the established composition of the CST complex (Figure 1B, Table S1). To validate this interaction, we co-expressed Flag-STN1 and HA-NPM1 in HEK 293T cells and confirmed their association through co-immunoprecipitation (Co-IP) (Figure 1C). To further dissect the interaction mechanism between NPM1 and CST, we performed GST pull-down assays and demonstrated that the acidic domain of NPM1 directly binds to the STN1 component of the CST complex (Figure 1D-F). Given NPM1's established role in DNA damage repair, this finding prompted us to further explore its functional significance in the ALT pathway.

### NPM1 plays an essential role in ALT-associated break-induced telomere replication (BITR)

In ALT cells, the majority of CST nuclear foci were reported to colocalize with telomeric DNA, consistent with their localization in APBs 23. Based on our observation that NPM1 interacts with the telomere-binding complex CST (STN1) in ALT cells, we sought to investigate whether NPM1 contributes to ALT telomere maintenance.

**Figure 2:**
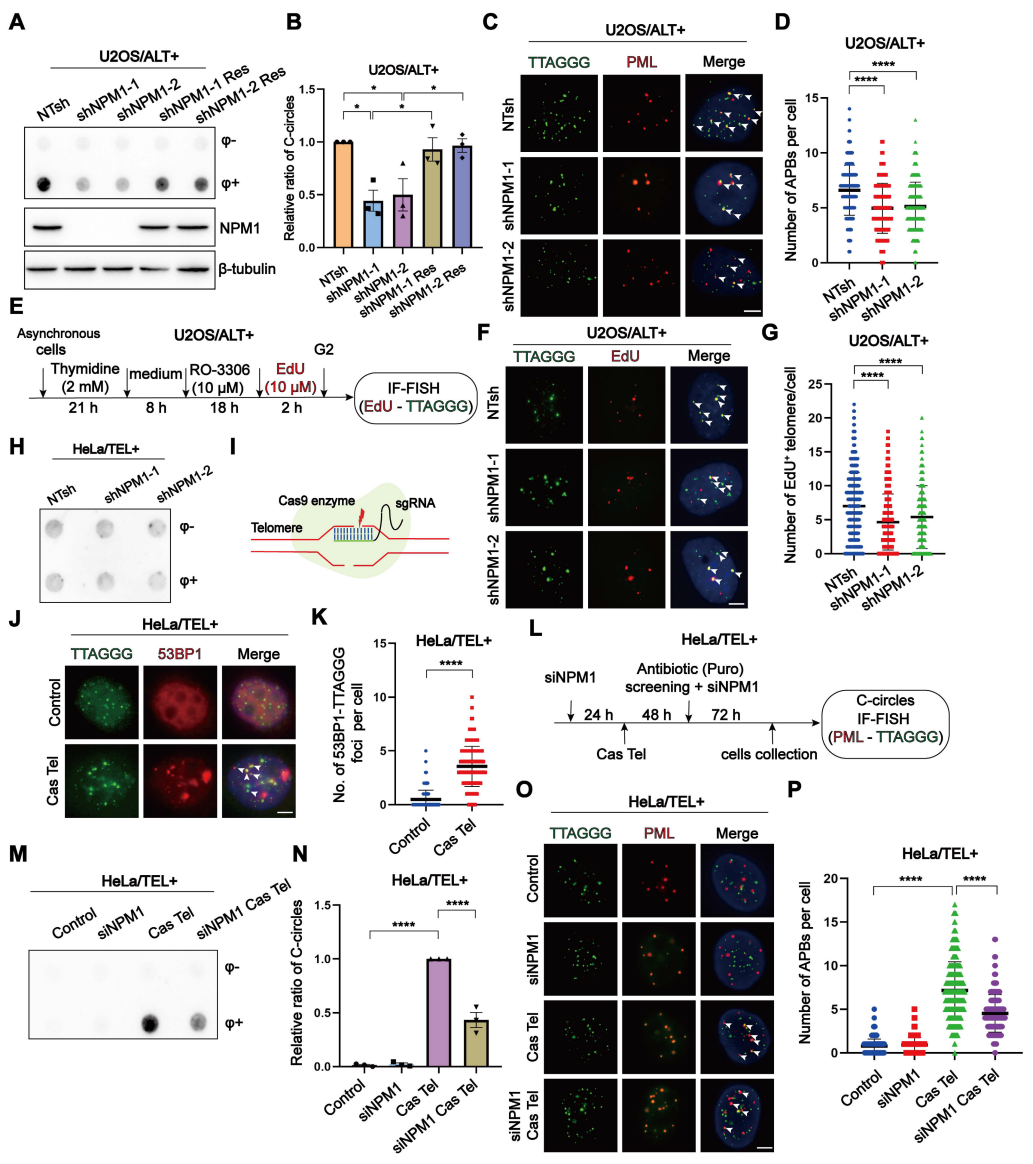
** NPM1 plays an essential role in ALT-associated break-induced telomere replication (BITR). (A-B)** C-circles and Western blotting analyses were performed in U2OS cells expressing non-target shRNA (NTsh), NPM1 knockdown (sh*NPM1*), and NPM1 rescue (sh*NPM1* Res) contructs, using 3 biological replicates. The relative levels of C-circles were quantified and shown in (B).** (C-D)** APBs formation was examined in the indicated U2OS cell clones, and co-localization of telomere and PML signals was analyzed by IF-FISH. The number of APBs per cell was quantified, with each dot representing the number of APBs in individual cells. 100 cells were counted for each condition.** (E)** Schematic representation of cell cycle synchronization and G2 phase telomere incorporation.** (F-G)** Telomere synthesis at G2 phase was analyzed by IF-FISH, with green indicating telomere and red indicating EdU. The co-localized signals were quantified in individual cells. 130 cells were counted for each condition.** (H)** C-circles analysis was performed in *NPM1* knockdown HeLa clones with 3 biological replicates. **(I)** Schematic representation of telomere-specific RNA-guided Cas9 nuclease. **(J-K)** Telomere damage induced by Cas Tel was determined by the co-localization of telomere (green) and 53BP1 (red), with quantification shown in (K). 100 nuclei were analyzed per condition from 3 independent experiments.** (L)** Schematic diagram of telomere damage by Cas Tel and *NPM1* knockdown (si*NPM1*) in HeLa cells. **(M-N)** C-circles were determined in the indicated HeLa cells with 3 biological replicates, and the relative levels of C-circles were quantified.** (O-P)** APBs were analyzed in the indicated conditions, and the number of APBs per nucleus was quantified. Each dot represents the number of APBs per nucleus in individual cells. 100 cells were counted for each condition. All data represent the mean ± SEM, n = 3 biological replicates.^ *^P<0.05, ^****^P<0.0001 (one-way ANOVA with Fisher's LSD tests).

Firstly, we disrupted NPM1 in U2OS cells using NPM1-targeting shRNA, resulting in the derivation of two knockdown clones (Figure S1A). Subsequently, we evaluated key hallmarks of ALT activity—including C-circles formation, ALT-associated PML bodies (APBs), and telomeric DNA synthesis—following *NPM1* depletion. Our findings illustrated that the absence of NPM1 resulted in a substantial decrease of about 50% in C-circles for U2OS cell lines. This effect was effectively reversed by re-expression of the shRNA-resistant WT NPM1 (Figure 2A-B). The phenomenon of reduced C-circles due to *NPM1* depletion was also corroborated in SaoS2, another ALT cell line (Figure S1B-D). Consistently, *NPM1* depletion reduced the number of APBs foci in U2OS cells by approximately 25% (Figure 2C-D). As G2 phase telomeric DNA synthesis represents the recombination replication occurring in ALT, we examined the telomere synthesis in G2 phase using EdU (5-ethynyl-2'-deoxyuridine) incorporation (Figure 2E, Figure S1E-F). The current data again demonstrated that *NPM1* depletion significantly decreased ALT telomere synthesis (Figure 2F-G). Telomeric mitotic DNA synthesis (MiDAS) is a conservative, break-induced replication (BIR)-like DNA synthesis process that plays a critical role in ALT-associated telomere replication 47-49. To investigate whether NPM1 is required for this process, we analyzed telomeric MiDAS in U2OS cells using an aphidicolin (APH)-induced replication stress model, which promotes accumulation of under-replicated telomeric DNA. Loss of NPM1 markedly reduced telomeric MiDAS events under both unstressed and APH-treated conditions (Figure S1G-I), indicating that NPM1 is essential for MiDAS in ALT cells. Consistent with impaired telomere replication, NPM1 depletion also led to increased telomere free ends (signal-free ends, SFEs) and fragile telomeres (multi-telomere signals, MTSs), further demonstrating elevated telomere instability in the absence of NPM1 (Figure S1J-L). Moreover, *NPM1* depletion significantly increased telomere dysfunction-induced foci (TIFs) in U2OS cells, indicating elevated telomere damage following *NPM1* knockdown (Figure S1M-N). These results collectively support a critical role of NPM1 in maintaining ALT telomere replication and integrity.

Given that the telomeric DNA damage triggers telomere recombination, ultimately leading to the development of ALT-like phenotypes via BITR in telomerase-positive cells 22, we next sought to elucidate the role of NPM1 in this process. First, we investigated whether *NPM1* depletion affects C-circles accumulation in ALT-negative HeLa cells. The data showed that *NPM1* knockdown had no impact on C-circle levels in these cells (Figure 2H), indicating that NPM1 is not essential for telomere maintenance in the absence of ALT activity. Next, we induced telomeric DSBs in HeLa cells using the Cas Tel system, which expresses the Cas9 together with a telomere-targeting sgRNA to specifically cleave telomeric regions (Figure 2I). This treatment activated telomere damage-induced repair pathways associated with BITR, as previously reported 20. Five days after Cas Tel expression, prominent telomere dysfunction-induced foci (TIFs) were readily detected, confirming efficient induction of telomeric DNA damage (Figure 2J-K). Importantly, Cas Tel-induced telomere damage led to a robust increase in C-circle levels and formation of APBs, hallmarks of ALT-like telomere activity. However, this accumulation was significantly mitigated upon *NPM1* depletion, indicating the pivotal involvement of NPM1 in BITR process in telomerase-positive cells (Figure 2L-P). Notably, treating cells with the ribonucleotide reductase inhibitor hydroxyurea (HU), which induces genomic DNA replication stress, did not affect the levels of C-circle (Figure S2A), indicating that global replication stress alone is insufficient to trigger BITR or ALT-like activity. Taken together, these findings suggest that NPM1 is required for telomeric DNA synthesis not only in ALT-positive cells, but also in telomerase-positive cells undergoing telomere-specific damage, supporting a broader role for NPM1 in DNA damage-induced telomere maintenance.

### NPM1 interacts with CST to maintain ALT-associated BITR

**Figure 3:**
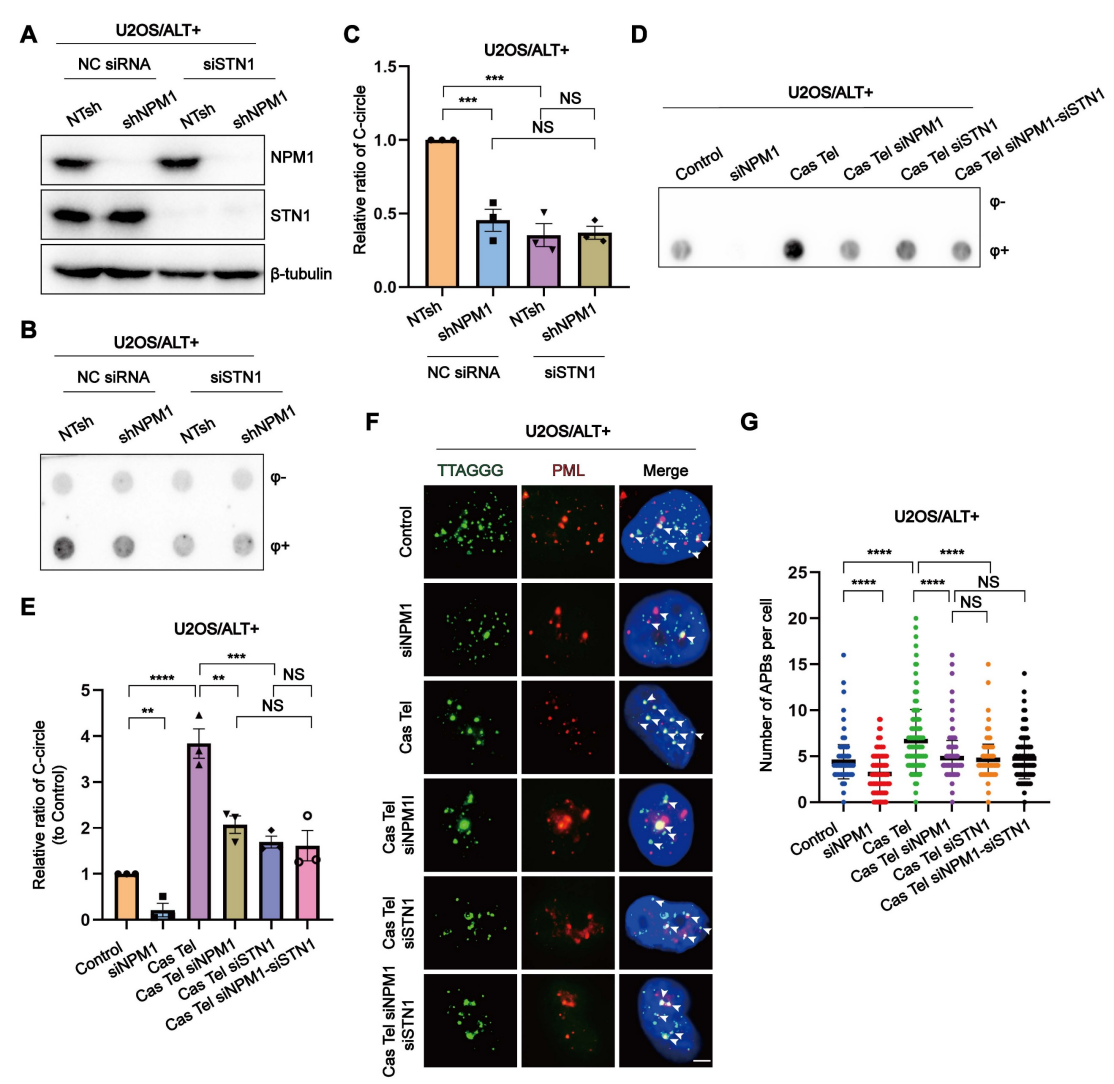
** NPM1 interacts with CST to maintain ALT-associated BITR. (A)** Western blot analysis showing the expression of NPM1 and STN1 in U2OS cells expressing either non-target shRNA (NTsh) or NPM1 shRNA, combined with negative control siRNA (NC) or siSTN1 treatment.** (B)** C-circles were analyzed in the indicated U2OS cells with 3 biological replicates. **(C)** The quantification of C-circles analysis shown from (B). **(D-E)** C-circles were analyzed in the indicated U2OS cells with 3 biological replicates, and the quantification is shown in (E).** (F-G)** The formation of APBs and quantification in representative cells. White arrows indicate co-localized signals. Each dot represents the number of APBs per nucleus in (G). 100 cells were counted for each condition. Data represent the mean ± SEM. n = 3 biological replicates. ^**^P<0.01, ^***^P<0.001, ^****^P<0.0001 (one-way ANOVA with Fisher's LSD tests).

Our discovery of the interaction between NPM1 and CST in ALT cells prompted us to further investigate their functional interplay. To this end, we generated single or double knockdowns of *NPM1* (shRNA) and *STN1* (siRNA) in U2OS and SaoS2 cell lines (Figure 3A, Figure S3A-C). Interestingly, in these ALT cell lines, the decrease in C-circles caused by NPM1 depletion was not further reduced by additional STN1 knockdown (Figure 3B-C, Figure S3D-E). We next examined the role of CST and NPM1 in the ALT-associated BITR model. In this context, NPM1 depletion significantly reduced the accumulation of both C-circles and APBs, and additional STN1 depletion did not lead to a further decrease beyond that observed with NPM1 knockdown alone (Figure 3D-G, Figure S3F-I). To further assess the contribution of NPM1 to ALT-mediated telomere maintenance within the CST pathway, we performed quantitative fluorescence *in situ* hybridization (Q-FISH) to analyze telomere length in ALT-positive cells. Q-FISH revealed that depletion of either NPM1 or STN1 alone significantly shortened telomeres; however, simultaneous knockdown of both genes did not further exacerbate telomere shortening (Figure S3J-K).

### The phosphorylation of Thr199-NPM1, possibly facilitated through the ATR signaling pathway, is essential for telomeric damage repair

**Figure 4:**
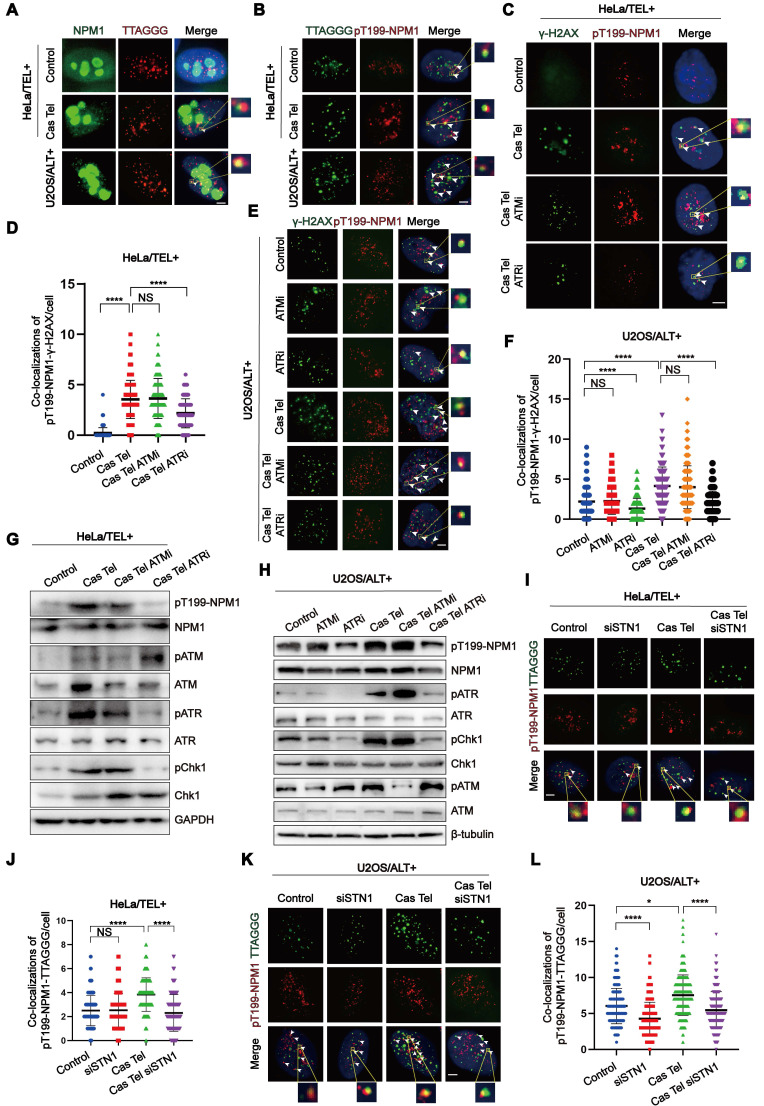
** The phosphorylation of Thr199-NPM1, possibly facilitated through ATR, is essential for telomeric damage repair. (A)** IF-FISH images showing co-localization of endogenous NPM1 (green) with TTAGGG (red) in representative cells.** (B)** IF-FISH images showing co-localization of TTAGGG (green) with pT199-NPM1 (red) in representative cells. **(C)** Co-localization of γ-H2AX (green) and pT199-NPM1 (red) was determined by immunofluorescence in cells treated with ATR inhibitor (ATRi, 10 μM) or ATM inhibitor (ATMi, 10 μM). White arrows indicate co-localized signals. **(D)** Quantification of co-localization between γ-H2AX and pT199-NPM1 in (C). 100 cells were counted for each condition.** (E)** Co-localization of γ-H2AX (green) and pT199-NPM1 (red) was determined by immunofluorescence in the indicated U2OS cell lines, White arrows indicate co-localized signals.** (F)** Quantification of co-localization between γ-H2AX and pT199-NPM1 in (E). 100 cells were counted for each condition.** (G-H)** Western blotting showing the expression of total and phosphorylated levels of ATR, ATM, Chk1, and NPM1 in HeLa and U2OS cells treated with ATRi (10 μM, 24 h), ATMi (10 μM, 24 h), or Cas Tel-induced telomere damage. **(I)** Representative images showing co-localization between pT199-NPM1 (red) and TTAGGG (green) in representative cells. White arrows indicate co-localized signals.** (J)** Quantification of co-localization between pT199-NPM1 and TTAGGG in (I). 80 cells were analyzed for each condition. **(K-L)** Co-localization of TTAGGG (green) with pT199-NPM1 (red) was determined in representative cells by IF-FISH. White arrows indicate co-localized signals. 100 cells were counted for each condition. Data represents the mean ± SEM. n = 3 biological replicates. ^*^P<0.05, ^****^P<0.0001 (one-way ANOVA with Fisher's LSD tests).

To investigate the role of NPM1 in ALT telomere synthesis and telomeric damage induced replication, we firstly examined its subcellular localization by IF. We found that, although a small fraction of NPM1 can leave the nucleolus and localize to telomeres or DNA damage sites, the majority remains strongly enriched in the nucleoli (Figure 4A, Figure S4A). Given that previous studies have implicated phosphorylated Thr199-NPM1 (pT199-NPM1) in the repair of irradiation-induced DSBs 39, we hypothesized that the NPM1 fraction colocalizing with telomeres upon damage might be its phosphorylated form. Accordingly, we performed IF-FISH using a pT199-NPM1 antibody together with telomere-specific PNA probes to assess their colocalization. In HeLa cells, Cas Tel treatment markedly increased the number of pT199-NPM1 foci associating with telomeres compared with untreated cells. Notably, U2OS cells displayed an even higher level of pT199-NPM1-telomere colocalization under basal conditions (Figure 4B, Figure S4B), suggesting that pT199-NPM1—rather than total NPM1—plays a functional role in telomeric DNA repair. The specificity of the pT199-NPM1 antibody was validated by both Western blotting and IF (Figure S4C-E). Notably, pT199-NPM1 signals were almost completely abolished after *NPM1* knockdown by shRNA, confirming the specificity of the anti-pT199-NPM1 antibody.

Cas Tel treatment can induce telomere-specific DNA damage and activate DNA damage response (DDR) pathways, potentially involving both ATR and ATM. Previous reports have shown that pT199-NPM1 can be phosphorylated by ATR and interact with downstream effectors such as Chk1 50. Based on this, we hypothesized that ATR and/or ATM signaling may regulate pT199-NPM1 recruitment to damaged sites. To further test this hypothesis, we examined the colocalization of pT199-NPM1 with γ-H2AX—a marker of DNA damage—in HeLa and U2OS cells treated with or without Cas Tel. We observed a robust recruitment of pT199-NPM1 to DNA damage foci following Cas Tel treatment. Treatment with a selective ATR inhibitor (ATRi), but not an ATM inhibitor (ATMi), significantly reduced the colocalization between pT199-NPM1 and γ-H2AX (Figure 4C-F), suggesting that the recruitment of pT199-NPM1 to damaged sites is primarily dependent on ATR signaling.

Given that telomeric DNA synthesis in ALT cells proceeds through a BITR process, we speculated that pT199-NPM1 might participate in this pathway. Compared with HeLa cells, U2OS cells showed higher levels of pT199-NPM1 recruitment to DNA damage sites, and this recruitment was markedly suppressed by ATR inhibition (Figure 4E-F). These findings further support a critical role for ATR in mediating pT199-NPM1 function in telomeric DNA repair, even in ALT cells in the absence of additional induced telomeric breaks. Consistently, Western blotting analysis demonstrated that ATR inhibition specifically reduced T199 phosphorylation of NPM1 in both HeLa and U2OS cells (Figure 4G-H), reinforcing the regulatory link between ATR signaling and pT199-NPM1 activity at damaged sites. These findings collectively suggest that the activation of pT199-NPM1 may be facilitated through ATR. From an alternative perspective, concerning ATR inhibitors as potential drugs for treating ALT tumors, one of its plausible targets could be phosphorylated NPM1, which has been confirmed to exert an anti-tumor effect in ALT tumors. Next, we determined whether the recruitment of pT199-NPM1 to the telomeres or damaged sites was dependent on the presence of CST. Our data demonstrated that the increase of pT199-NPM1 at the telomeres or damaged sites induced by Cas Tel treatment was significantly reduced by *STN1* knockdown (Figure 4I-L, Figure S4F-G).

To more directly determine whether phosphorylated NPM1 is spatially associated with major ALT components, we examined its localization relative to both APBs and RAD52. Following Cas Tel-induced telomeric DNA breaks, three-color IF-FISH (pT199-NPM1, telomeric PNA, and PML) revealed a significant increase in colocalization between pT199-NPM1 and APBs in ALT cells (Figure S5A-B). Consistently, we also observed a pronounced increase in colocalization between pT199-NPM1 and RAD52 after Cas Tel treatment (Figure S5C-D), indicating that pT199-NPM1 may recruit to RAD52-marked recombination sites in response to telomere damage. To assess the functional relationship between NPM1/CST and RAD52, we performed G2/M telomeric DNA synthesis analyses using combinatorial knockdowns (siNC, siNPM1 or siSTN1, siRAD52, and double knockdowns). As expected, depletion of NPM1, STN1 or RAD52 alone led to a marked reduction in EdU incorporation at G2/M phase. Importantly, additional depletion of RAD52 did not further exacerbate these ALT defects beyond those observed with single NPM1 or STN1 knockdowns (Figure S5E-H), suggesting that NPM1/CST and RAD52 act within the same ALT repair pathway.

In summary, our findings support a crucial role for phosphorylated NPM1 in telomere damage repair and ALT telomere maintenance, acting through ATR-dependent phosphorylation and CST-mediated enrichment to telomeres and DNA damage sites, where it cooperates with RAD52 and APBs to promote ALT-associated BITR.

### The phosphorylation of Thr199 is required for NPM1 to maintain ALT process

**Figure 5:**
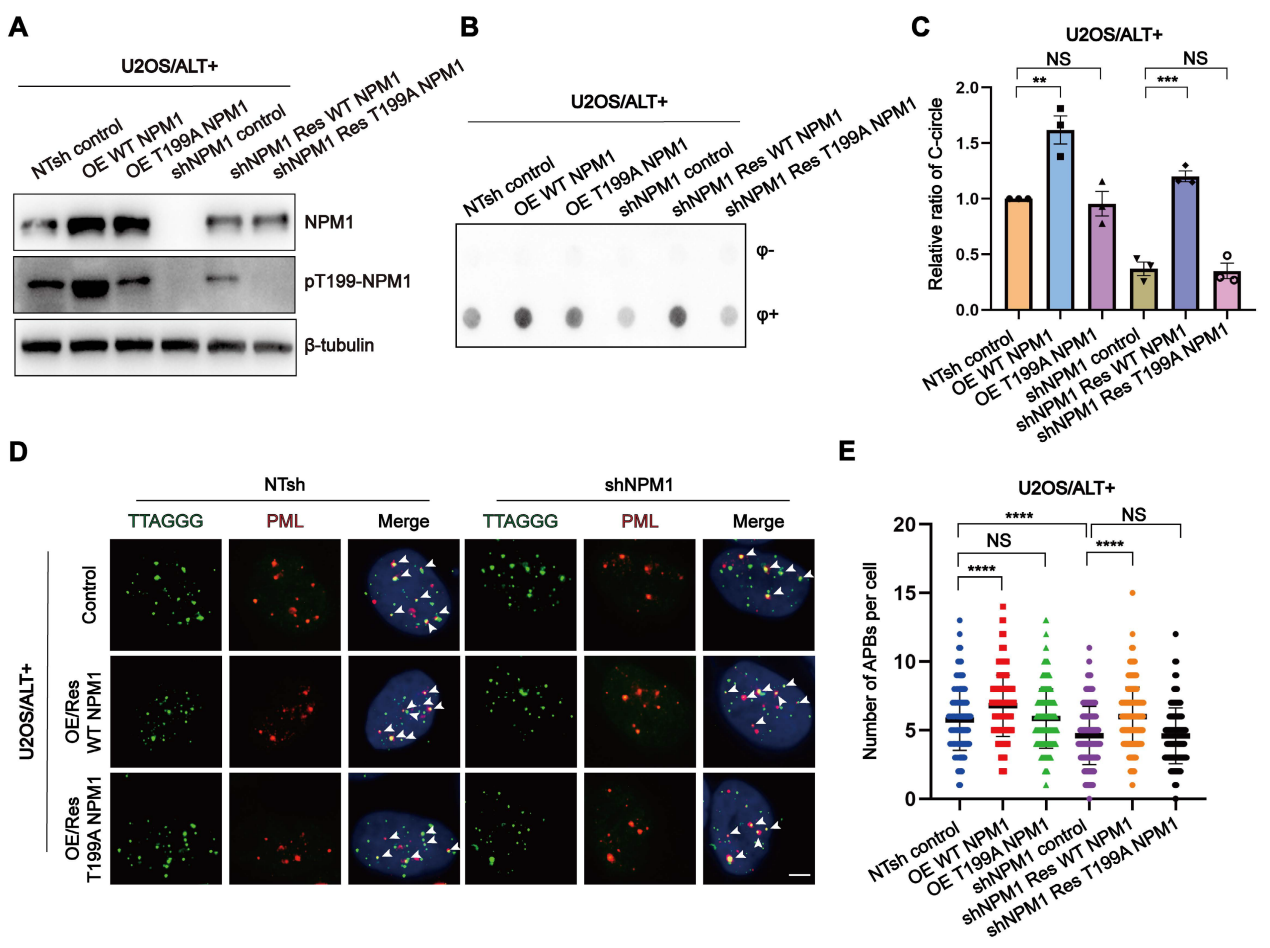
** The phosphorylation of Thr199 is required for NPM1 to maintain ALT process. (A)** Western blotting showing NPM1 and pT199-NPM1 expression in U2OS non-target shRNA (NTsh) and sh*NPM1* cells, and their corresponding wild-type (WT) or T199A NPM1 overexpression (OE WT/T199A NPM1) or rescue (sh*NPM1* Res WT/T199A NPM1) clones.** (B-C)** The levels of C-circles were detected in the indicated cell lines, with 3 biological replicates. Quantification is shown in (C). **(D)** The formation of APBs was determined by IF-FISH with telomere probe (green) and anti-PML antibodies (red). White arrows indicate co-localized signals. **(E)** The data from (D) were quantified and plotted. 100 cells were analyzed for each condition. n = 3 biological replicates. Data represents the mean ± SEM. n = 3 biological replicates. ^*^P<0.05, ^**^P<0.01, ^***^P<0.001, ^****^P<0.0001. One-way ANOVA with Fisher's LSD tests was used for (C and E).

To further investigate the role of Thr199 phosphorylation in NPM1-mediated ALT maintenance, we constructed shRNA-resistant wild-type (WT) and non-phosphorylatable mutant (T199A) NPM1 plasmids and transfected them into both NTsh and sh*NPM1* U2OS cell clones. NPM1-positive cells were sorted based on GFP co-expression (Figure S6A), and the successful overexpression was confirmed by Western blotting (Figure 5A, Figure S6B-C). Functional assays revealed that *NPM1* depletion led to a marked reduction in C-circles abundance and APBs formation. Notably, while WT NPM1 successfully rescued these effects, the T199A mutant failed to do so (Figure 5B-E), indicating that Thr199 phosphorylation is critical for NPM1's role in maintaining the ALT phenotype.

### Elevated pT199-NPM1 expression in osteosarcoma correlates with ALT activity and poor prognosis

**Figure 6:**
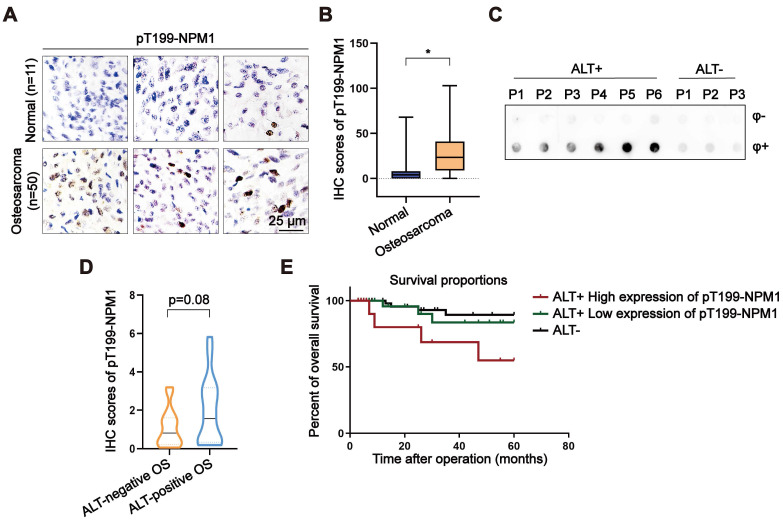
** Elevated pT199-NPM1 expression in osteosarcoma correlates with ALT activity and poor prognosis. (A-B)** Immunohistochemistry (IHC) was performed in normal bone and OS tissues. Shown are representative images (A) of pT199-NPM1 staining with IHC scores (B). **(C)** C-circle assay performed on OS patient samples to determine ALT status. Individual patients are labeled numerically (P1, P2, P3...), with ALT-positive and ALT-negative groups indicated accordingly. **(D)** Immunohistochemistry (IHC) was performed in ALT-positive or ALT-negative OS patients. Shown is the IHC scores for pT199-NPM1 staining.** (E)** Kaplan-Meier curves showing overall survival of ALT-positive OS patients (n = 17) with high versus low expression of pT199-NPM1. Log-rank test, *P* = 0.07, Hazard Ratio (logrank) = 3.576. Data represents the mean ± SD. ^*^P<0.05. Two tailed t test was used for (B and D).

Given that around 60% of OS relies on ALT for telomere maintenance, we assessed the expression of pT199-NPM1 in a tissue microarray (TMA) comprising OS and adjacent normal bone tissues. Immunohistochemistry (IHC) revealed a significant increase in pT199-NPM1 levels in tumor tissues compared to normal controls (Figure 6A-B). These findings suggest a potential association between elevated pT199-NPM1 and ALT activity.

To further investigate the clinical relevance of pT199-NPM1, a total of 33 clinical OS samples were collected and analyzed. ALT status was determined by C-circle assay, identifying 17 ALT-positive and 16 ALT-negative cases (Figure 6C, Figure S7A). IHC analysis showed that ALT-positive tumors exhibited higher levels of pT199-NPM1 compared to ALT-negative counterparts (Figure 6D). Among the ALT-positive group, patients were stratified into high (n = 7) and low (n = 10) pT199-NPM1 expression groups (Figure S7B). Kaplan-Meier survival analysis revealed that patients with higher pT199-NPM1 expression had significantly poorer overall survival (Figure 6E). These results suggest that pT199-NPM1 may serve as a potential prognostic biomarker in ALT-positive OS, although validation in larger cohorts is needed to confirm its predictive value and biological relevance.

### pT199-NPM1 mediates ALT maintenance by recruiting and stabilizing POLD3 on telomere

**Figure 7:**
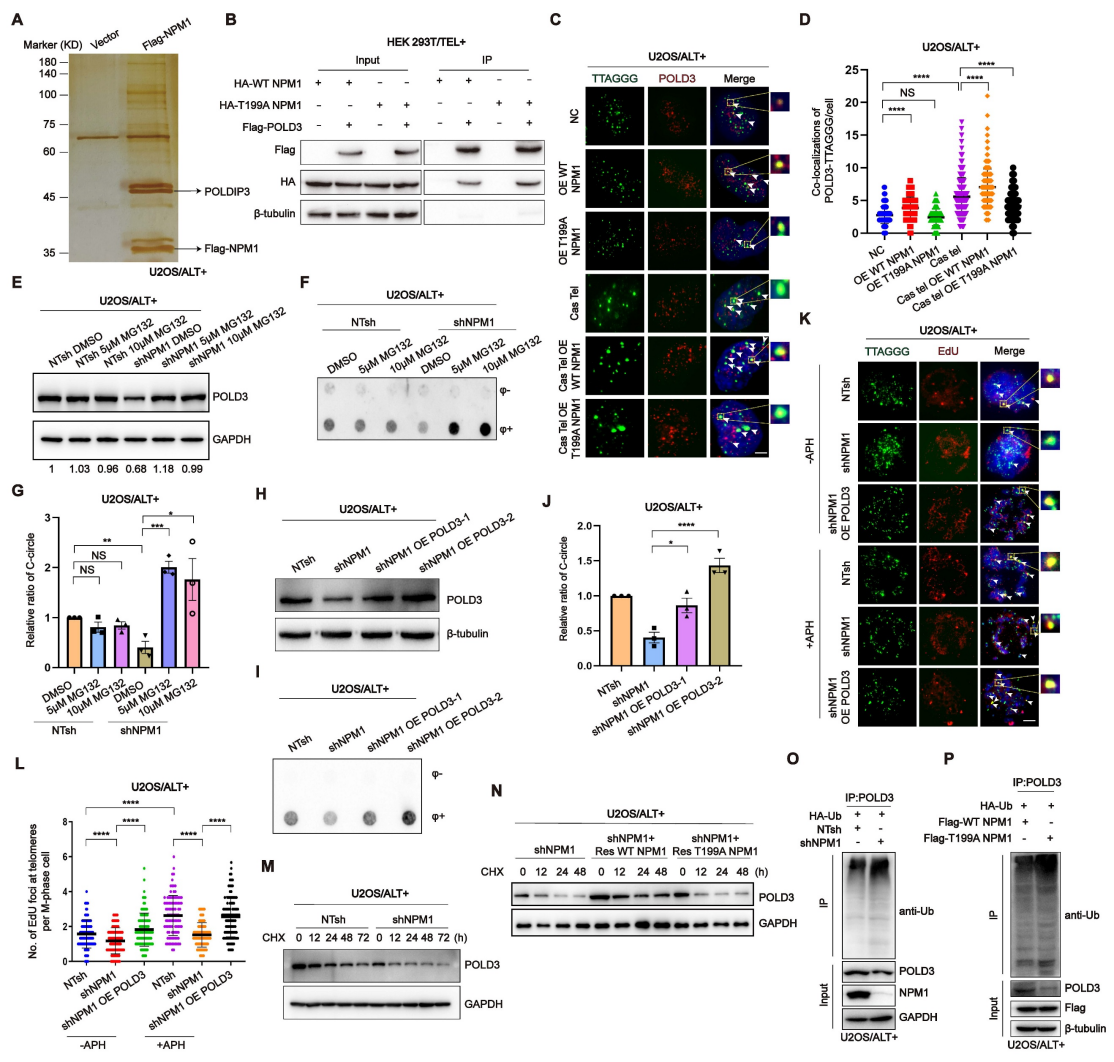
** pT199-NPM1 mediates ALT maintenance by recruiting and stabilizing POLD3 on telomere. (A)** HEK 293T cells were transfected with Flag-NPM1 for 48 h, followed by anti-Flag IP. Eluted proteins were analyzed by SDS-PAGE and visualized by silver staining.** (B)** Interaction between HA-WT-NPM1 or HA-T199A-NPM1 with Flag-POLD3 was analyzed by immunoprecipitation and Western blotting in HEK 293T cells. **(C-D)** Co-localization of POLD3 (red) with TTAGGG (green) was determined in representative U2OS cells by IF-FISH. White arrows indicate co-localized signals. 100 cells were counted for each condition.** (E)** Western blotting analysis of POLD3 expression in U2OS NT shRNA and *shNPM1* clones treated with the proteasome inhibitor MG132 for 24 h. β-tubulin was used as a loading control.** (F-G)** C-circles were analyzed in U2OS non-target shRNA (NTsh) and sh*NPM1* clones treated with 5 or 10 μM MG132 for 24 h, with 3 biological replicates. **(H)** Flag-*POLD3* was transfected into U2OS sh*NPM1* clone for 48 h. Western blotting analysis of POLD3 expression in the indicated cell lines. **(I)** C-circles analysis was performed on U2OS non-target shRNA (NTsh) and *NPM1* shRNA clones transfected with Flag-POLD3 for 48 h, with 3 biological replicates.** (J)** Quantification of relative levels of C-circles from (I).** (K-L)** Telomeric mitotic DNA synthesis (MiDAS) analysis of indicated cells with or without APH treatment. (K) Representative images of mitotic DNA synthesis at telomeres on metaphase spreads. (L) Quantification of telomeric MiDAS and the number of EdU-positive telomeres per metaphase spread. A total of 100 cells were analyzed for each condition.** (M)** U2OS non-target shRNA (NTsh) and sh*NPM1* cells were treated with CHX (100 µM) for 0, 12, 24, 48, and 72h. POLD3 protein levels were assessed by Western blotting, with GAPDH used as a loading control. **(N)** In U2OS sh*NPM1* cells, WT or T199A NPM1 was reintroduced. After CHX treatment for 0, 12, 24, and 48 h, POLD3 protein levels were assessed by Western blotting. **(O)** In U2OS non-target shRNA (NTsh) and sh*NPM1* cells, HA-Ub was overexpressed, and POLD3 was immunoprecipitated using an endogenous POLD3 antibody. The interaction between POLD3 and ubiquitin was analyzed by Western blotting. **(P)** U2OS cells were co-transfected with HA-Ub and either WT NPM1 or T199A NPM1. POLD3 was immunoprecipitated using an endogenous POLD3 antibody, and the interaction between POLD3 and ubiquitin was analyzed by Western blotting. Data is represented as the mean ± SEM from 3 biological replicates. Statistical significance was calculated using one-way ANOVA with Fisher's LSD tests:^ *^P<0.05, ^**^P<0.01, ^***^P<0.001, ^****^P<0.0001.

To investigate the mechanism underlying the role of NPM1 in ALT telomere DNA synthesis, we conducted immunoprecipitation-mass spectrometry to identify potential co-factors that interact with NPM1. The complete list of NPM1 binding proteins identified in this study is provided in table S2. Our analysis revealed that DNA Pol δ interacting protein 3 (POLDIP3) was among the list of binding proteins (Figure 7A). Considering that ALT telomere synthesis is likely mediated by BITR, which requires DNA Pol δ subunit POLD3/4 22, we hypothesized that the role of NPM1 in ALT telomere DNA synthesis might involve its interaction with the DNA polymerase POLD3. Our subsequent Co-IP experiments confirmed that NPM1 indeed interacts with POLD3 (Figure 7B). To further determine whether the binding between NPM1 and POLD3 is dependent on NPM1 phosphorylation, we examined the interaction between POLD3 and both WT NPM1 and T199A (mutant) NPM1. Surprisingly, our data showed that the phosphorylation of Thr199 is not required for the binding of NPM1 to POLD3, as the T199A NPM1 mutant still interacted with POLD3 (Figure 7B). To determine how NPM1 phosphorylation plays a role in modulating POLD3, we performed IF assays in U2OS cells. Following Cas Tel-induced telomeric DNA damage, POLD3 was increasingly recruited to telomeres. However, this recruitment was significantly reduced upon overexpression of the dominant-negative T199A NPM1 mutant, which cannot be phosphorylated at Thr199 (Figure 7C-D). Consistent results were also observed in telomerase-positive HeLa and HEK 293T cells (Figure S8A-D). These findings collectively indicate that Thr199 phosphorylation of NPM1 is essential for facilitating the localization of POLD3 to sites of telomeric damage.

As NPM1 has been reported to stabilize its binding partners by acting as a competitive E3 substrate 51, we investigated whether NPM1 could stabilize POLD3 on damaged telomere sites by inhibiting its proteasomal degradation. Our results showed that *NPM1* depletion caused the degradation of POLD3, which was restored by the addition of the proteasome inhibitor MG132 (Figure 7E). Moreover, the decrease in C-circles resulting from *NPM1* disruption was rescued by MG132 treatment (Figure 7F-G), indicating that NPM1 stabilizes POLD3 at damaged telomeres by preventing its proteasomal degradation, thereby facilitating ALT-mediated telomere synthesis. To validate whether NPM1 regulates ALT activity through POLD3, we overexpressed POLD3 in NPM1-depleted cells and assessed C-circle formation and telomeric MiDAS. POLD3 overexpression rescued the reduction in C-circles caused by NPM1 depletion (Figure 7H-J). Under the same conditions, NPM1 depletion markedly reduced telomeric MiDAS events in ALT cells both in the presence and absence of aphidicolin (APH), whereas POLD3 overexpression restored the MiDAS defect induced by NPM1 depletion (Figure 7K-L), indicating that NPM1 promotes ALT-associated telomeric DNA synthesis through a POLD3-dependent mechanism. To further evaluate the impact of NPM1 on POLD3 stability, we treated U2OS NTsh (non-targeting shRNA) and sh*NPM1* cells with cycloheximide (CHX) to inhibit new protein synthesis, and collected cell samples at different time points (0, 12, 24, 48, 72 h). Western blotting and quantitative analysis of CHX chase experiments showed that POLD3 degradation was significantly accelerated upon *NPM1* knockdown, resulting in a markedly shortened POLD3 half-life in NPM1-depleted cells (Figure 7M, Figure S8E), confirming that NPM1 is crucial for maintaining POLD3 stability. Next, we investigated whether the phosphorylation modification of NPM1 is essential for this process. In U2OS sh*NPM1* cells, we overexpressed WT and T199A NPM1, respectively. After CHX treatment, the results showed that reintroducing WT, but not T199A NPM1, could rescue the POLD3 protein instability caused by NPM1 knockdown (Figure 7N). Then we aimed to explore whether pT199-NPM1 stabilizes POLD3 protein levels through deubiquitination. Co-IP results revealed that *NPM1* knockdown in U2OS cells significantly increased the ubiquitination levels of POLD3 (Figure 7O). Moreover, in U2OS cells co-transfected with HA-Ub and either Flag-WT NPM1 or Flag-T199A NPM1, the Co-IP results showed that WT NPM1 overexpression markedly reduced POLD3 ubiquitination, whereas the T199A mutant failed to do so (Figure 7P). These findings confirm that pT199-NPM1 stabilizes POLD3 by promoting its deubiquitination, leading to the promotion of ALT telomere synthesis.

### Targeting NPM1 sensitizes doxorubicin treatment in ALT-positive osteosarcoma

**Figure 8:**
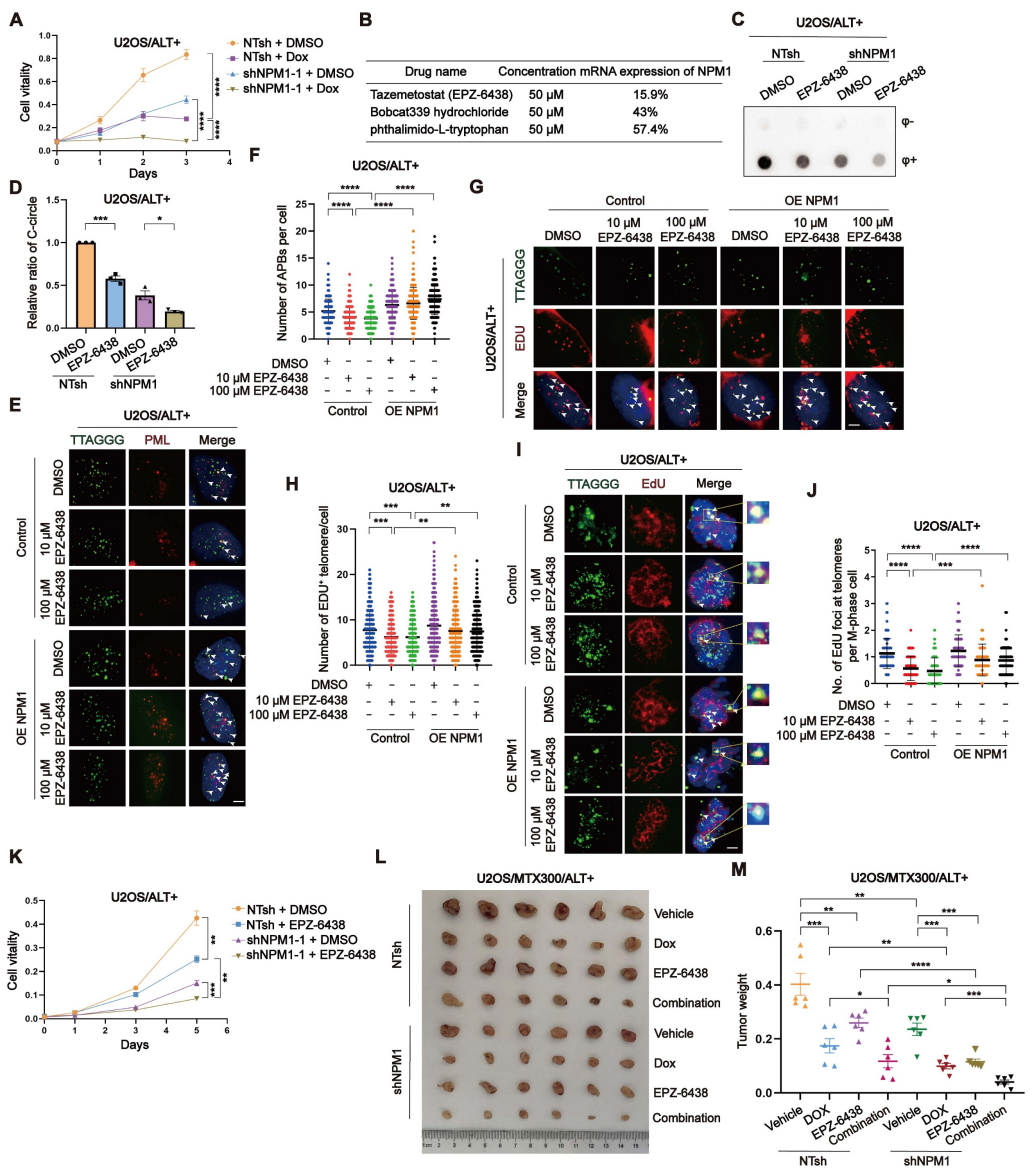
** Targeting NPM1 sensitizes doxorubicin treatment in ALT-positive osteosarcoma. (A)** Growth curve determined by CCK-8 assay. U2OS non-target shRNA (NTsh) and sh*NPM1* clones were treated with DMSO or Dox (0.075 μM) for 3 days and the absorbance of OD_450nm_ was detected.** (B)** The table shows the efficiency of the selected drugs in inhibiting *NPM1* mRNA expression.** (C-D)** C-circles assay was performed on U2OS non-target shRNA (NTsh) and sh*NPM1* clones treated with 10 μM EPZ-6438 for 3 days, with 3 biological replicates. The relative levels of C-circles from (C) were quantified. **(E-F)** APBs were analyzed in the indicated conditions, and the number of APBs per nucleus was quantified. Each dot represents the number of APBs per nucleus in individual cells. 100 cells were counted for each condition.** (G-H)** Telomere synthesis at G2 phase was analyzed by IF-FISH, with green indicating telomere and red indicating EDU. The co-localized signals were quantified in individual cells. 120 cells were counted for each condition (n = 2 biological replicates).** (I-J)** MiDAS analysis in the indicated U2OS cells. (I) Representative images showing mitotic DNA synthesis at telomeres on metaphase spreads. (J) Quantification of telomeric MiDAS and number of EdU-positive telomeres per metaphase spread. 100 cells were counted for each condition. **(K)** CCK-8 assay of non-target shRNA (NTsh) and sh*NPM1* clones in U2OS cells treated with DMSO or EPZ-6438 (10 μM) for 5 days. **(L-M)** U2OS/MTX300 xenograft growth assays in BALB/c nude mice received vehicle, Dox (7.5 mg/kg) (twice a day for 2 weeks; intravenous), EPZ-6438 (200mg/kg) (twice a day for 25 days; oral gavage), or both. Tumor weights were measured at end point. Data represents the mean ± SEM. n = 3 biological replicates. ^*^P<0.05, ^**^P<0.01,^ ***^P<0.001, ^****^P<0.0001. Two tailed t test was used for (A and I). One-way ANOVA with Fisher's LSD tests was used for (D, F and H).

Doxorubicin (Dox) is a commonly used first-line chemotherapy drug for OS treatment. Its mechanism of action involves intercalating with DNA and inhibiting topoisomerase II, leading to DNA damage and apoptosis of cancer cells 52. However, Dox also has several limitations, including dose-dependent toxicity, drug resistance 53. To investigate the potential therapeutic benefits of targeting NPM1 in ALT-positive tumors, we first examined the effect of *NPM1* depletion on cell survival in ALT-positive U2OS/SaoS2 and ALT-negative HEK 293T cells. The results showed that *NPM1* depletion resulted in a significant reduction in cell viability in ALT-positive cells, while the effect was much less pronounced in HEK 293T cells (Figure 8A, Figure S9A-B). We then assessed the combinatorial effects of *NPM1* knockdown and Dox treatment on U2OS cells proliferation using CCK-8 assay. Our data revealed that *NPM1* depletion impairs U2OS cell proliferation and significantly increases the sensitivity of cells to Dox treatment (Figure 8A, Figure S9C).

Based on the above findings, we hypothesized that NPM1 suppression could sensitize ALT-positive OS cells to Dox treatment. To explore potential NPM1-targeting compounds, we conducted a high-throughput screening assay using the PerkinElmer Opera Phenix system and two commercial compound libraries, including Histone Modifcation Research Compound Library and Transcription Factors Library. Among the top candidates, tazemetostat (EPZ-6438), a selective EZH2 inhibitor; Bobcat339 hydrochloride, a selective TET enzyme inhibitor that modulates DNA demethylation; and phthalimido-L-tryptophan, a compound involved in tryptophan metabolism, demonstrated the highest transcriptional inhibitory effects (Figure 8B). The complete screening results, including all tested compounds that exhibited > 10% inhibition efficiency for *NPM1* transcription and their relative effects, are provided in table S3. Subsequently, we conducted comprehensive experiments in culture and* in vivo* experiments to assess the efficacy of EPZ-6438, a prominent epigenetic drug employed in the treatment of epithelioid sarcoma, in inhibiting NPM1. While the specific mechanism of EPZ-6438's action on NPM1 remains unclear, our findings confirm its effectiveness in our experimental models. Firstly, we treated the ALT-positive OS cells and telomerase-positive HeLa cells with EPZ-6438 and measured the mRNA transcription and protein expression of NPM1. Our results indicated that EPZ-6438 treatment led to a reduction of NPM1 (Figure S9D-E). To preliminarily assess whether this effect extends to other genes involved in ALT, we also examined the expression of *BRCA1*, *BRCA2*, and *RAD51* following EPZ-6438 treatment. These genes did not exhibit significant changes in mRNA expression (Figure S9F-H), suggesting that EPZ-6438 does not broadly affect the transcription of these tested genes in the context of ALT. However, this does not exclude the possibility that it may regulate other genes, which warrants further investigation in future studies. Consistent with NPM1 suppression, C-circles formation was reduced after EPZ-6438 treatment (Figure 8C-D, Figure S9I-J). Moreover, we observed a significant decrease in APBs formation, G2 phase telomeric DNA synthesis, and conservative telomeric MiDAS in U2OS cells, which was restored upon NPM1 re-expression (Figure 8E-J, Figure S9K-L). As a result, we observed that EPZ-6438 treatment inhibited the proliferation of ALT-positive cells, but not that of telomerase-positive HeLa cells (Figure 8K, Figure S9M-N).

However, the observation that EPZ-6438 also inhibited the proliferation of sh*NPM1* clones (Figure 8K, Figure S9M-N) may be attributable to multiple factors. One possible explanation is that *NPM1* knockdown by shRNA was incomplete, and EPZ-6438 may further suppress residual NPM1 expression at the transcriptional level, thereby exacerbating the destabilization of downstream targets such as POLD3 and further impairing ALT activity. Secondly, the anti-tumor effects of EPZ-6438 may not be solely mediated through NPM1 suppression. As a known EZH2 inhibitor, EPZ-6438 may exert additional anti-tumor activities via epigenetic regulation or through other signaling pathways.

To assess the effectiveness of combining Dox and EPZ-6438* in vivo*, we implanted U2OS/MTX300 cells, a methotrexate (MTX)-resistant derivative of U2OS cells, into BALB/c nude mice and subjected them to various treatment regimens. Our findings revealed that while Dox and EPZ-6438 administered individually led to tumor suppression, their combination significantly enhanced the inhibition of tumor growth (Figure 8L-M). Taken together, although EPZ-6438 reduces NPM1 expression and disrupts ALT-associated phenotypes, its continued efficacy in NPM1-deficient cells suggests it may exert additional anti-tumor effects through NPM1-independent pathways. These findings highlight the therapeutic potential of EPZ-6438, particularly when used in combination with Dox, in treating ALT-positive OS.

## Discussion

In this study, a novel activity of NPM1 was identified as being associated with CST and playing a crucial role in ALT. Our findings have demonstrated that the phosphorylation of NPM1 at Thr199, mediated by the ATR signaling pathway, stabilizes POLD3 and protects it from proteasomal degradation, thereby maintaining the ALT telomeres. Furthermore, we have identified a small molecule compound EPZ-6438 that inhibits NPM1 transcription and translation, blocks ALT activity, and sensitizes Dox treatment in OS therapy in mouse xenograft model. Taken together, our data reveal a novel telomere damage repair signaling pathway (CST/pT199-NPM1/POLD3) that is critical for homologous directed telomere replication and ALT cells survival. Our findings show new light on the mechanism of telomere maintenance in ALT cells and provide a potential therapeutic strategy for targeting ALT-positive tumors.

Building upon the established model that CST complex facilitates C-circle generation by priming DNA synthesis at lagging-strand telomeres 54, 55, our study adds a critical regulatory layer to this process. We demonstrate that telomere damage triggers ATR-dependent phosphorylation of NPM1 at Thr199, and that CST may require for the efficient recruitment of pT199-NPM1 to damaged telomeres. At these sites, pT199-NPM1 supports the stabilization of ALT-associated POLD3 by preventing its ubiquitin-mediated degradation. This regulation ensures efficient C-circle production and G2/M telomeric DNA incorporation, thereby providing a more comprehensive mechanistic understanding of how this hallmark ALT activity is sustained in cancer cells.

Interestingly, our observations do not fully align with those of a previous study 23, which reported that stable shRNA-mediated STN1 knockdown in U2OS cells did not affect APB levels or global telomere length under unstressed conditions. In our experiments, however, transient siRNA-mediated STN1 depletion led to a reduction in APB numbers even in untreated ALT cells, and APB levels were further decreased following Cas Tel-induced telomeric DNA damage. Moreover, high-resolution Q-FISH analysis revealed that STN1 knockdown also caused telomere shortening, which may have been difficult to detect by TRF analysis in the previous study. Together, these findings support a context-dependent role of STN1 in ALT telomere maintenance, particularly under conditions of telomeric DNA damage or replication stress.

**Figure 9:**
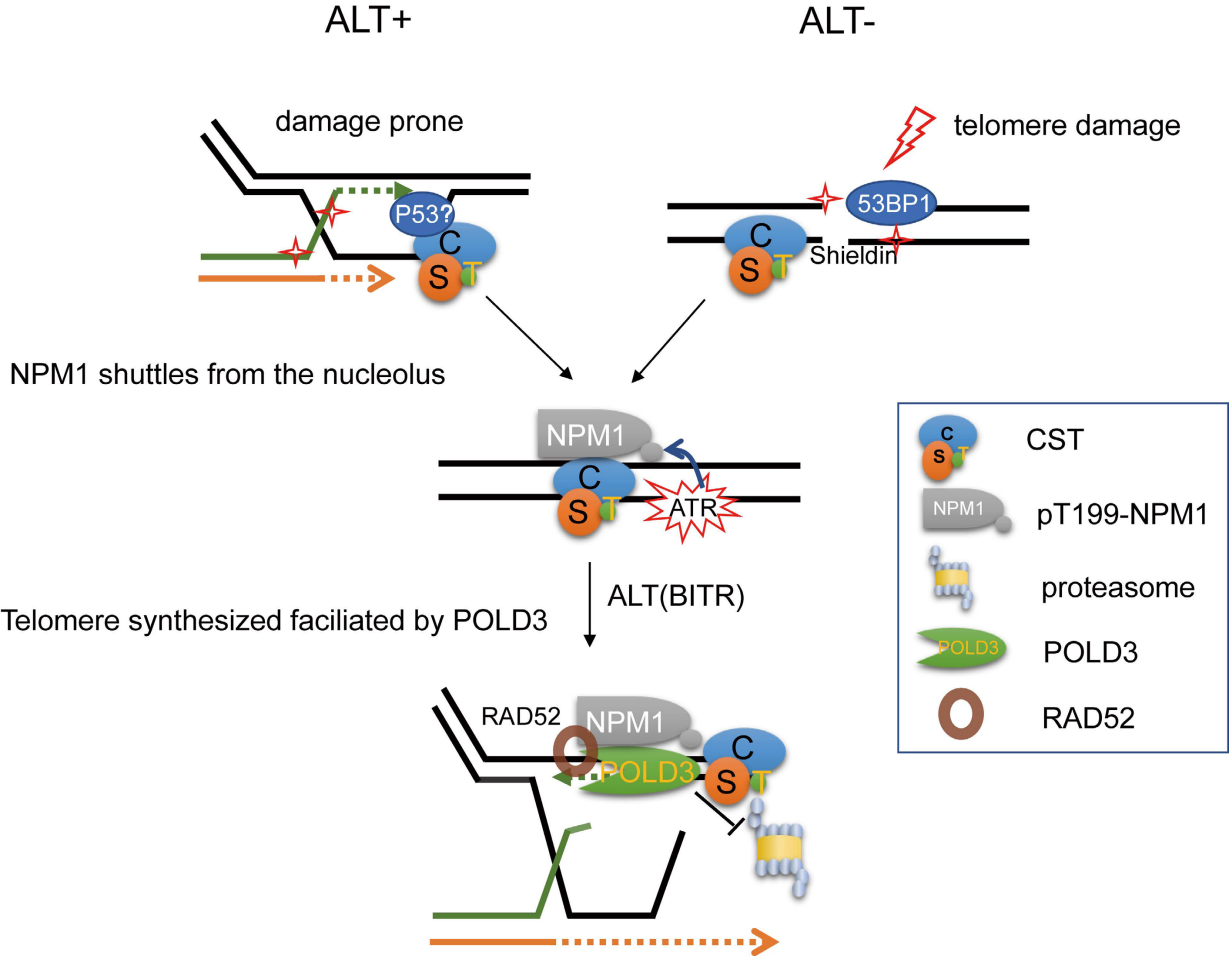
** Proposed model for the maintenance of ALT telomere by NPM1/CST/POLD3 complex.** In ALT-positive cells, telomeres are intrinsically damage-prone and frequently undergo break-induced telomere replication (BITR). CST plays an essential role by priming DNA synthesis, thereby generating C-rich intermediates that serve as precursors for C-circle formation. Upon telomere damage, CST (CTC1-STN1-TEN1) recruits NPM1 from the nucleolus to damaged telomeres, where ATR signaling phosphorylates NPM1 at Thr199. This pT199-NPM1 then cooperates with CST to stabilize the ALT effector POLD3 by preventing its ubiquitin-proteasome-mediated degradation, thereby sustaining POLD3 at damaged telomeres to support poly δ-dependent telomeric DNA synthesis and maintain ALT activity.

ALT-positive cells are considered telomeric damage-prone and thus undergo BITR. Upon this condition, DNA repair factors such as p53, 53BP1, and the telomere-associated CST complex are recruited to the damaged sites. During subsequent telomere repair, CST recruits NPM1 and promotes its phosphorylation via ATR signaling. Phosphorylated NPM1 then stabilizes POLD3, thereby facilitating ALT-dependent telomere DNA synthesis. Similarly, in ALT-negative cells, when telomeric double-strand breaks occur, DNA damage markers such as 53BP1 and the shelterin complex are recruited to the lesion. These factors, in turn, facilitate CST complex recruitment, which subsequently brings phosphorylated NPM1 to the damaged telomeres to participate in telomeric repair (Figure 9).

Previous studies have suggested that NPM1 protein is highly expressed in various tumor tissues and plays a significant role in cancer cell survival 30, 34. However, its involvement in ALT tumors has not been previously reported. Our IHC data show that pT199-NPM1 is highly expressed in ALT-positive OS. Although this cannot directly confirm whether the observed pT199-NPM1 is specifically involved in telomere damage repair or ALT maintenance, its elevated expression in ALT-positive OS and its correlation with shorter overall survival suggest that pT199-NPM1 may play a broader role in tumor progression, potentially involving mechanisms related to telomeres. Moreover, our data indicate that NPM1 is required for telomere maintenance following the induction of telomeric DNA breaks, a condition that robustly activates ALT-associated BITR. We propose that telomeric DNA damage generates a strong demand for efficient repair and DNA synthesis at chromosome ends, and that pT199-NPM1 is essential for supporting this ALT-associated repair process and tumor cell maintenance.

While previous studies suggest that blocking ALT signals alone may not sufficiently inhibit the growth of ALT-positive cells in the short term, our findings indicate that *NPM1* depletion leads to significant telomere damage and shortening, as evidenced by decreased telomere fluorescence unit (TFU) and increased TIFs formation. These effects are likely to induce cumulative stress over time, which could explain the significant impact on cell viability observed in our experiments. Additionally, NPM1 plays a crucial role in DNA damage repair pathways beyond ALT regulation, contributing to its broader effects on cell survival. Therefore, the impact of *NPM1* depletion on ALT-positive cells may not only be due to its inhibition of ALT, but also involves its role in maintaining telomeres integrity and genomic stability.

Telomere maintenance is crucial for cell survival, and homologous recombination directed telomere synthesis plays a critical role in telomere repair and replication 6. We observed that ATR stimulated the phosphorylation of Thr199-NPM1 on telomeric DSBs sites and that POLD3, a component of DNA poly δ essential for break-induced DNA replication was involved. Notably, ATR inhibition specifically disrupted ALT activity and selectively triggered apoptosis in ALT tumors 56. We propose that ATR inhibitor suppressed the Thr199 phosphorylation of NPM1 and disrupted the POLD3 on telomere, ultimately leading to telomere dysfunction and cellular apoptosis. Notably, NPM1 could interact and be activated by Chk1 and CDK, which function downstream of the ATR signal 27, 50, 57. This suggests that NPM1 may not be a direct target of ATR but could be activated indirectly during telomere damage repair processes.

Furthermore, our study revealed a novel pathway where CST coats damaged telomeres, recruits NPM1, and stabilizes POLD3 with the help of P53/ATR, ultimately facilitating telomere synthesis for ALT cancer cells. However, chemotherapy-induced DNA damage can also affect telomeres, making NPM1 a potential therapeutic target for telomerase-positive cancers. Targeting NPM1 may impair proper telomere synthesis which could potentially enhance the efficacy of chemotherapy and improve overall survival rates for patients.

We also found that EZH2 inhibitor EPZ-6438 combined with Dox significantly inhibits the growth of ALT tumors, although the exact mechanism by which EPZ-6438 inhibits NPM1 requires further investigation. EZH2 is an important epigenetic regulatory factor that alters chromatin structure by methylation of lysine 27 of histone H3 (H3K27me3), thereby affecting gene transcriptional regulation 58. We speculate that EZH2-H3K27me3 may promote the transcriptional levels of *NPM1*, thereby facilitating the growth of ALT-positive OS. In summary, our findings provide new insights into the molecular mechanisms underlying ALT telomere maintenance and have important implications for the development of targeted therapies for NPM1.

## Supplementary Material

Supplementary figures and tables.
