# Supplementary material for: NPM1 phosphorylation-mediated telomere maintenance via stabilization of POLD3 in ALT-positive osteosarcoma: unraveling mechanisms and therapeutic opportunities
Source: Theranostics. 2026 Jan 22;16(8):4224–44. doi: 10.7150/thno.108662 (PMC12905828; doi:10.7150/thno.108662)
Supplement: Supplementary file 1 — Supplementary figures and tables. [file thnov16p4224s1.pdf]

Figure S1

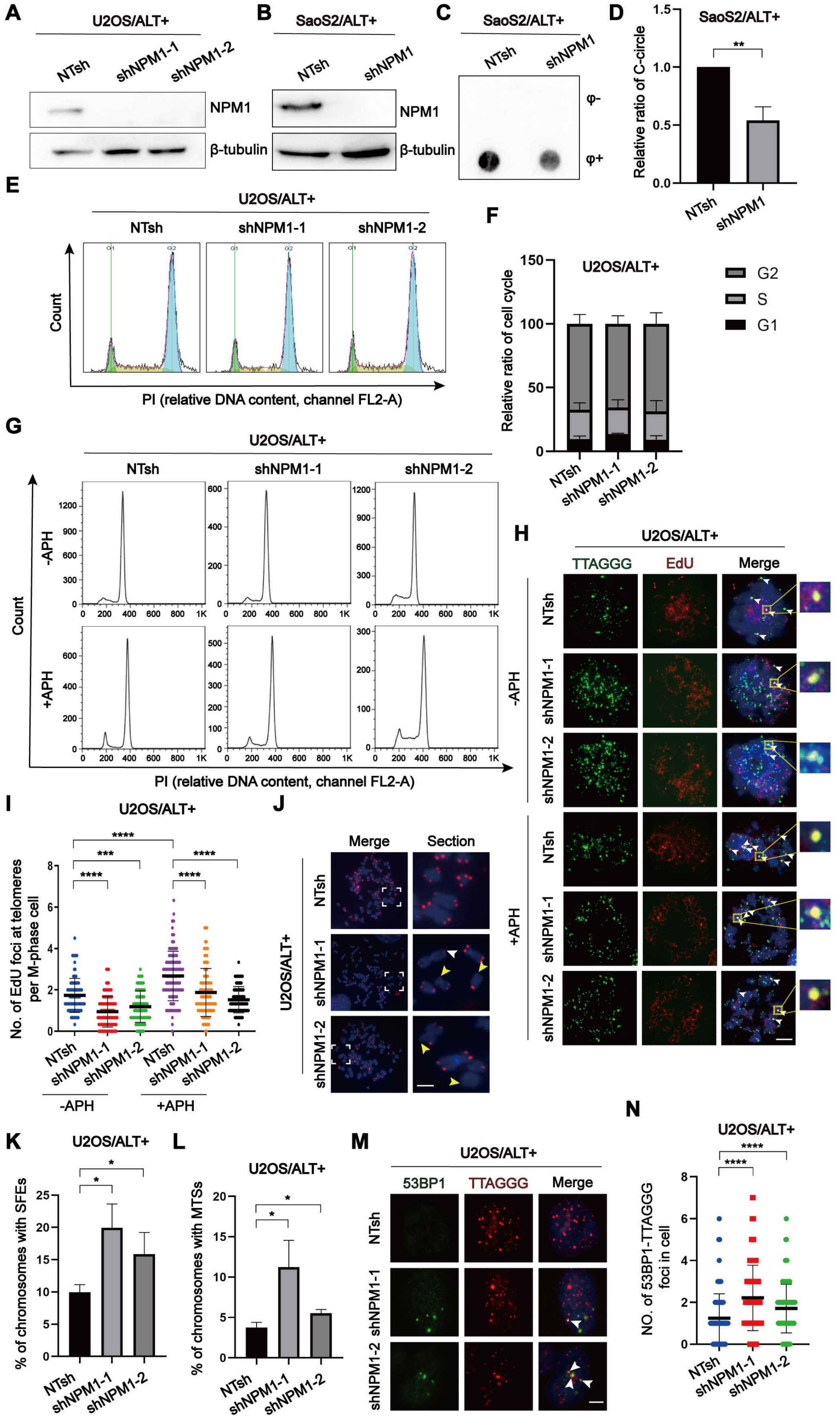
A
U2OS/ALT+
NTsh
shNPM1-1
shNPM1-2
NPM1
β-tubulin
B
SaoS2/ALT+
NTsh
shNPM1
NPM1
β-tubulin
C
SaoS2/ALT+
NTsh
shNPM1
φ-
φ+
D
SaoS2/ALT+
Relative ratio of C-circle
NTsh
shNPM1
E
U2OS/ALT+
NTsh
shNPM1-1
shNPM1-2
Count
PI (relative DNA content, channel FL2-A)
F
U2OS/ALT+
Relative ratio of cell cycle
G2
S
G1
G
U2OS/ALT+
-APH
+APH
Count
PI (relative DNA content, channel FL2-A)
H
U2OS/ALT+
TTAGGG
EdU
Merge
I
U2OS/ALT+
No. of EdU foci at telomeres per M-phase cell
-APH
+APH
J
Merge
Section
U2OS/ALT+
K
U2OS/ALT+
% of chromosomes with SFEs
L
U2OS/ALT+
% of chromosomes with MTSs
M
U2OS/ALT+
53BP1
TTAGGG
Merge
N
U2OS/ALT+
NO. of 53BP1-TTAGGG foci in cell

Figure S1. Depletion of NPM1 impaired ALT activity.

(A) Western blotting of non-targeting (NT) and *NPM1* knockdown (sh*NPM1*) clones in U2OS cells with NPM1 antibody. **(B)** Western blotting of NT shRNA and NPM1 knockdown (sh*NPM1*) cells in SaoS2 with NPM1 antibody. **(C-D)** C-circles analysis in SaoS2 with NT and NPM1 shRNA cells. The relative levels of C-circles were quantified in (D). **(E)** Flow cytometry assay showing that U2OS cell cycle arrest in G2/M phase. **(F)** Cell cycle profiles of U2OS NT shRNA and sh*NPM1* clones. **(G)** Flow cytometry assay showing that U2OS cell cycle arrest in G2/M phase. **(H-I)** Telomeric mitotic DNA synthesis (MiDAS) analysis of U2OS NTsh and shNPM1 cells with or without APH treatment. (H) Representative images showing mitotic DNA synthesis at telomeres on metaphase spreads. (I) Quantification of telomeric MiDAS and number of EdU-positive telomeres per metaphase spread. 100 cells were counted for each condition. **(J)** Telomere FISH on metaphase spreads from U2OS cells in the indicated conditions. Examples of aberrant telomeres are shown in section images. Yellow arrows indicate signal-free ends (SFEs) and white arrows indicate multi-telomere signals (MTSs). **(K-L)** Quantification of the percentage of chromosomes with SFEs and MTSs. More than 600 chromosomes were analyzed in each experiment. **(M-N)** IF-FISH images and quantification showing 53BP1 (green) co-localization with telomeres (red) (telomere dysfunction-induced foci, TIFs) in the indicated cell lines (n = 100). All data represents the mean ± SD. n = 3 experiments. $^{*}P<0.05$, $^{**}P<0.01$, $^{****}P<0.0001$. Two-tailed t-test was used for (D). One-way ANOVA with Fisher's LSD tests was used for (I, K, L and N).

Figure S2

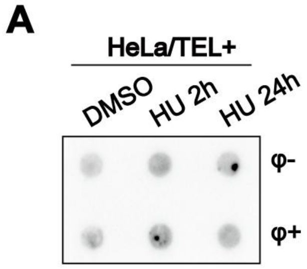

Figure S2. The treatment with HU does not affect the level of C-circles.

(A) After treating the HeLa cells with DMSO or HU (2mM) for 2 hours or 24 hours, collect the cells to measure the level of C-circles.

Figure S3

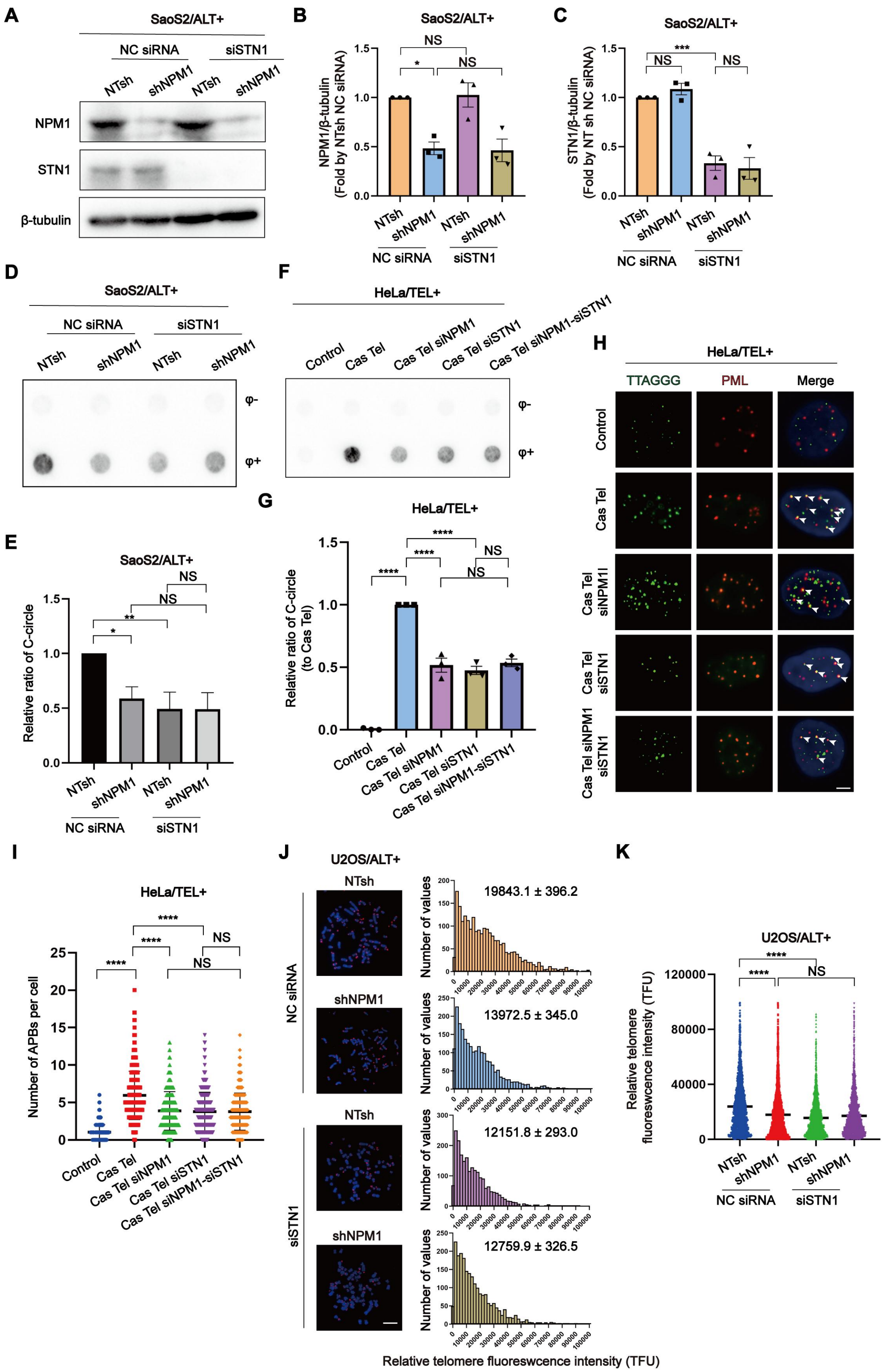
A
SaoS2/ALT+
NC siRNA
siSTN1
NTsh
shNPM1
NPM1
STN1
β-tubulin
B
SaoS2/ALT+
NPM1/β-tubulin (Fold by NTsh NC siRNA)
NS
*
C
SaoS2/ALT+
STN1/β-tubulin (Fold by NT sh NC siRNA)
***
D
SaoS2/ALT+
φ-
φ+
E
SaoS2/ALT+
Relative ratio of C-circle
**
F
HeLa/TEL+
Control
Cas Tel
Cas Tel siNPM1
Cas Tel siSTN1
Cas Tel siNPM1-siSTN1
G
HeLa/TEL+
Relative ratio of C-circle (to Cas Tel)
****
H
HeLa/TEL+
TTAGGG
PML
Merge
I
HeLa/TEL+
Number of APBs per cell
J
U2OS/ALT+
Number of values
19843.1 ± 396.2
13972.5 ± 345.0
12151.8 ± 293.0
12759.9 ± 326.5
Relative telomere fluoreswcence intensity (TFU)
K
U2OS/ALT+
Relative telomere fluoreswcence intensity (TFU)

Figure S3. NPM1 promotes the maintenance of ALT by interacting with CST.

(A) Western blot analysis showing the expression of NPM1 and STN1 in SaoS2 cells expressing either non-target shRNA (NTsh) or NPM1 shRNA, combined with negative control siRNA (NC) or siSTN1 treatment. β-tubulin served as loading control. **(B-C)** Quantification of NPM1 and STN1 protein levels as shown in (A). **(D)** C-circles analysis in the indicated cell lines. **(E)** Data from (D) were quantified. **(F-G)** C-circles were analyzed in the indicated HeLa cells with 3 biological replicates, and the quantification is shown in (G). **(H-I)** The formation of APBs and quantification in representative cells. White arrows indicate co-localized signals. Each dot represents the number of APBs per nucleus in (I). 100 cells were counted for each condition. **(J)** Representative telomere Q-FISH images in U2OS non-target control (NC), *NPM1* sh knock-down, *STN1* si knock-down and *NPM1*/*STN1* double knock-down cells. Telomere were labeled telomere PNA probes (red), and chromosomed were labeled with DAPI (blue). Histogram showed distribution of relative telomere length displayed as fluorescence intensity (TFU, telomere fluorescence unit). **(K)** Quantification of telomere fluorescence units (TFU) from Q-FISH analysis shown in (J). Approximately 600 chromosome ends in total were analyzed. Data represents the mean ± SEM. n = 3 biological replicates. $^{*}P$<0.05, $^{**}P$<0.01, $^{***}P$<0.001, $^{****}P$<0.0001 (one-way ANOVA with Fisher's LSD tests).

Figure S4

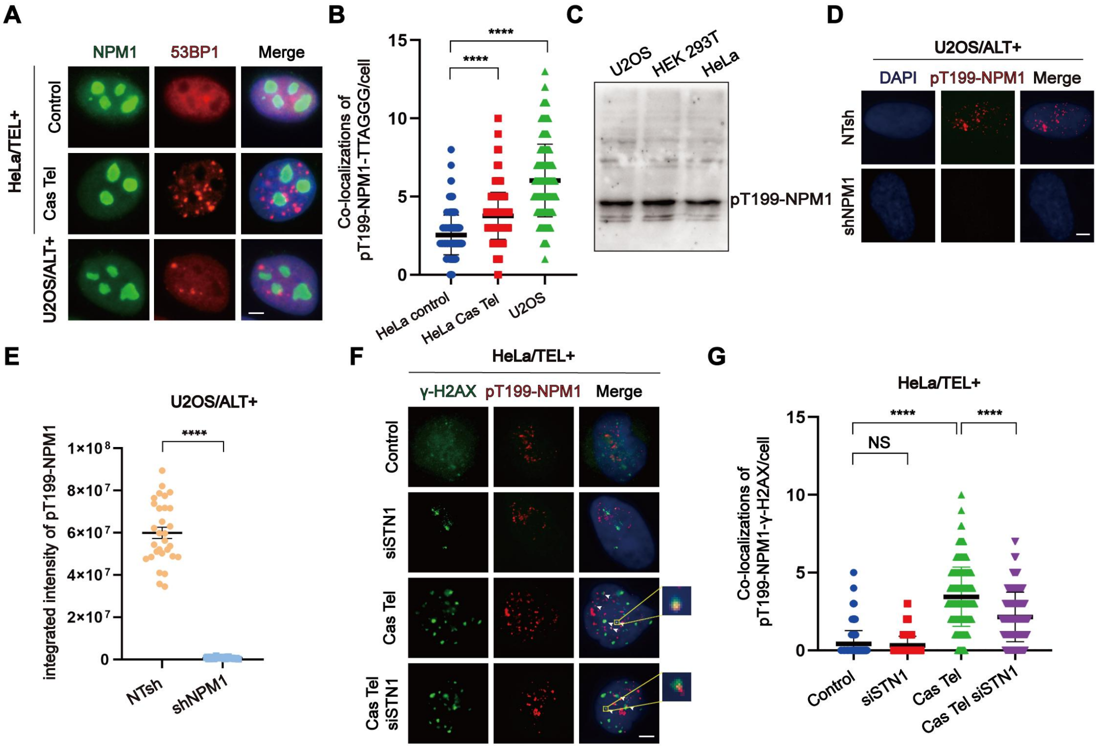

Figure S4. Recruitment of pT199-NPM1 to injury sites is dependent on CST.

(A) IF was carried out using anti-NPM1 (green) and 53BP1 (red) antibodies in representative cells. **(B)** Quantification of co-localization between pT199-NPM1 and TTAGGG in U2OS and HeLa cells treated with Cas Tel, based on the IF images shown in Figure 4B. 100 cells were counted for each condition. **(C)** Western blotting analysis of pT199-NPM1 antibody specificity. A full membrane was probed with pT199-NPM1 antibody to assess its specificity. The blot shows a distinct band corresponding to the expected molecular weight of NPM1, confirming the antibody's specificity. Additional bands represent non-specific binding or degradation products. **(D)** IF results show the expression of pT199-NPM1 foci in U2OS NTsh and sh*NPM*1 cells. **(E)** Quantification of the integrated fluorescence intensity of pT199-NPM1 from (D). For each group, 10 images were analyzed per replicate, with each image containing at least 10 nuclei and representing the total fluorescence intensity of all nuclei in the field. The experiment was performed in 3 independent biological replicates. **(F-G)** IF images and quantification showing pT199-NPM1 co-localization with γ-H2AX in the indicated cell lines (n = 100). Data represents the mean ± SEM. n = 3 biological replicates. $^{*}P<0.05$, $^{****}P<0.0001$. One-way ANOVA with Fisher's LSD tests was used for (B and G). Two tailed t test was used for (E).

Figure S5

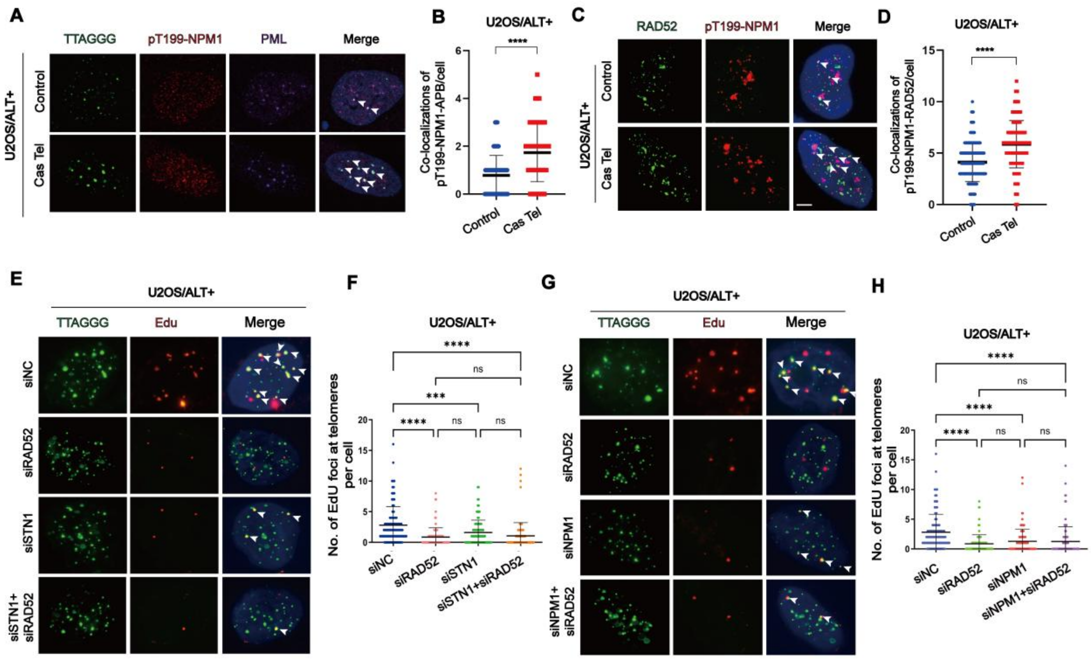

Figure S5. pT199-NPM1 is recruited to RAD52 and APBs foci upon telomere damage and maintains ALT markers independently of RAD52.

(A-B) Three-color IF-FISH showing pT199-NPM1, telomeres, and PML (APBs) in representative cells. The data from (C) were quantified and plotted. 100 cells were analyzed for each condition. **(C)** IF images showing co-localization of RAD52 (green) with pT199-NPM1 (red) in representative cells. **(D)** Quantification of co-localization between RAD52 and pT199-NPM1 in (C). 100 cells were counted for each condition. **(E and G)** Telomere synthesis at G2 phase was analyzed by IF-FISH, with green indicating telomere and red indicating EdU. White arrows indicate co-localized signals. The co-localized signals were quantified in individual cells. **(F and H)** 100 cells were counted for each condition. All data represents the mean ± SD. *P<0.05, **P<0.01, ***P<0.001, ****P<0.0001. Two-tailed t-test was used for (B and D). One-way ANOVA with Fisher's LSD tests was used for (F and H).

Figure S6

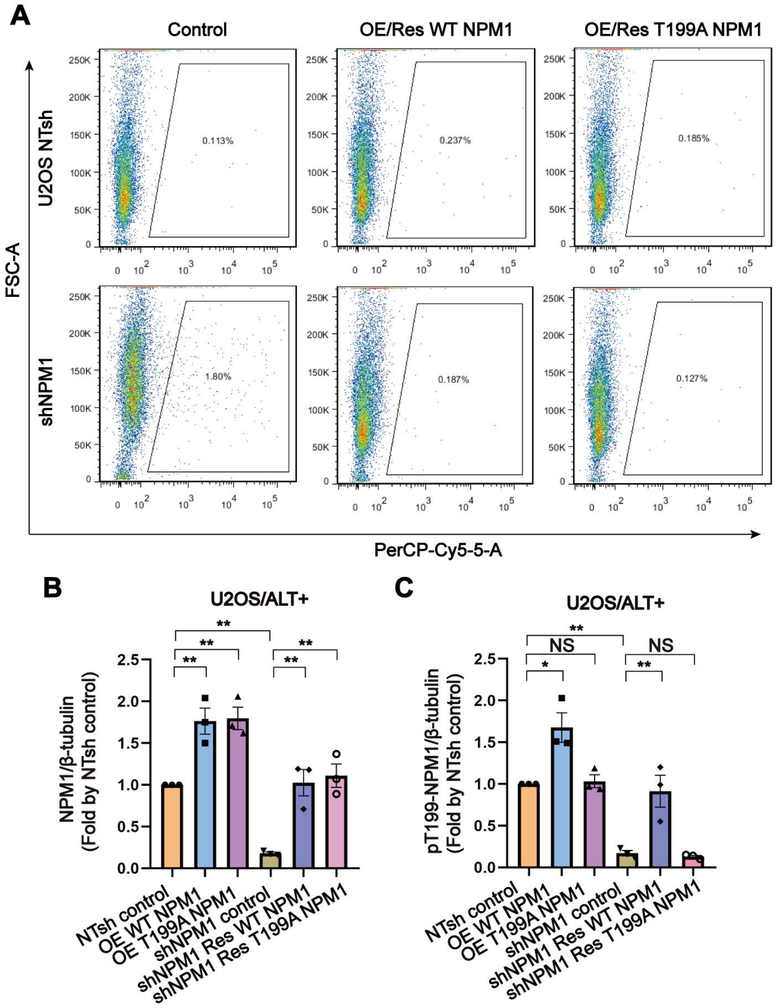

Figure S6. Enrichment and expression validation of NPM1 and pT199-NPM1.

(A) Flow cytometry assay showing the proportion of cells overexpressing WT NPM1 and T199A NPM1 in U2OS NT shRNA and sh*NPM1* clones. **(B-C)** Quantification of NPM1 and pT199-NPM1 protein levels as shown in Figure 5A.

Figure S7

A

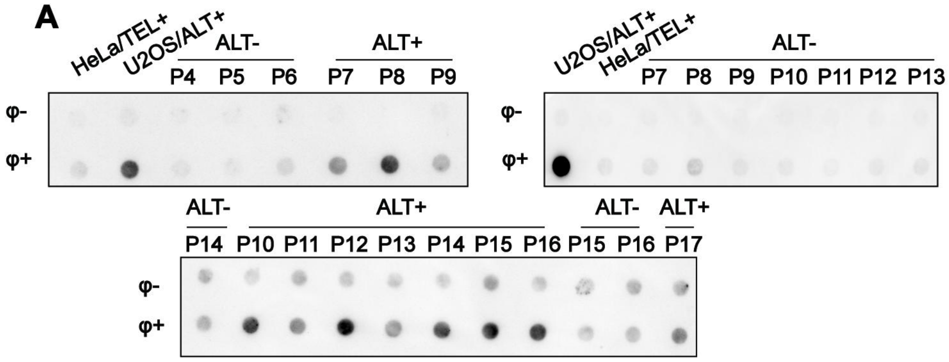

B

	ALT+			ALT-		
Gender	number of patients	Death	%	number of patients	Death	%
Male	10	4	40.0	9	2	22.2
Female	7	3	42.9	7	1	14.3
Grade	number of patients	Death	%	number of patients	Death	%
I	13	4	30.8	10	1	10.0
II	3	2	66.7	4	1	25.0
III	1	1	100.0	2	1	50.0
pT199-NPM1	number of patients	Death	%	number of patients	Death	%
High expression	7	4	57.1	2	1	50.0
Low expression	10	3	30.0	14	2	14.3

Figure S7. High expression of pT199-NPM1 was positively correlated with reduced survival in patients with ALT- positive OS.

(A) C-circle assay analysis of ALT activity in individual OS patient samples. Each dot represents one patient and is labeled numerically. **(B)** The table displays the number of patients diagnosed with both ALT-positive and ALT-negative OS, along with their gender, OS grading, and mortality status.

Figure S8

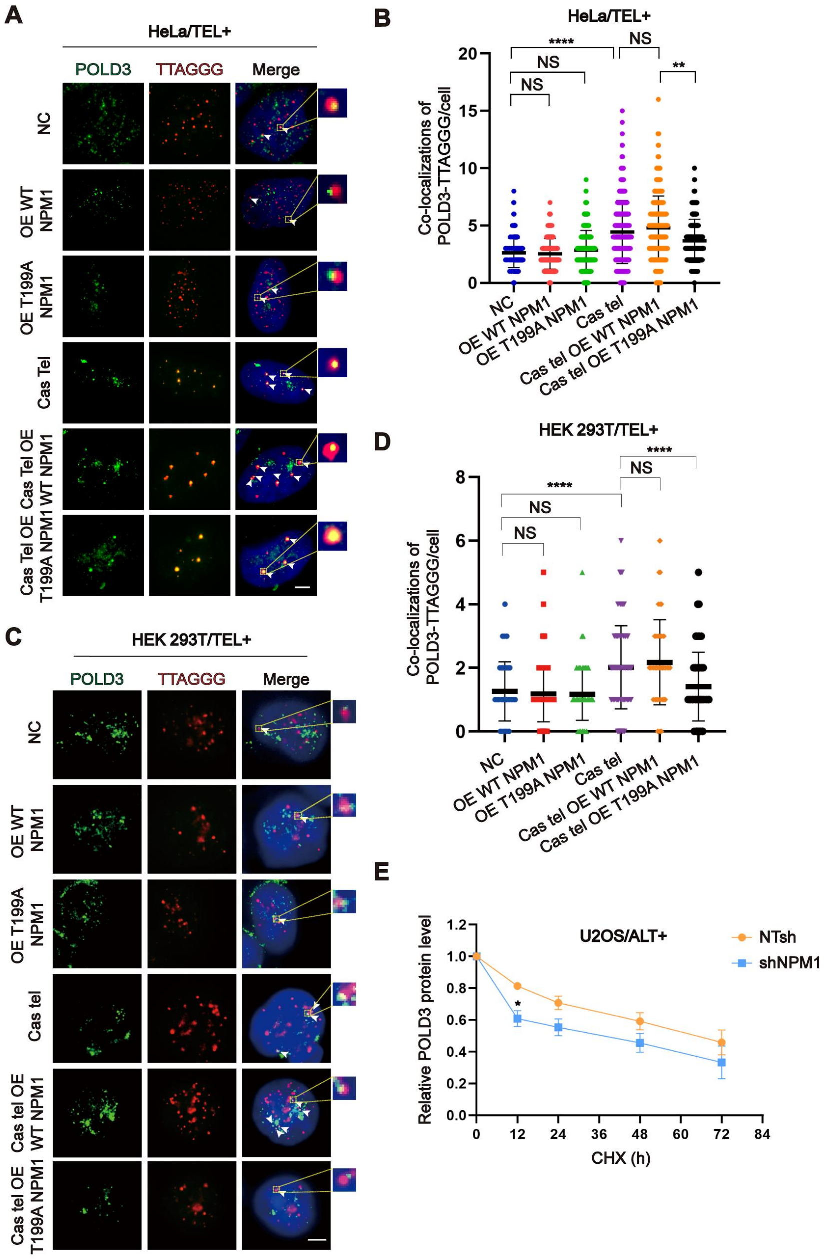

Figure S8. pT199-NPM1 stabilizes POLD3 on telomere in both ALT-positive U2OS cells and telomerase-positive HEK 293T cells.

(A) Co-localization of POLD3 (green) with TTAGGG (red) was determined in representative HeLa cells by IF-FISH. White arrows indicate co-localized signals. **(B)** The data from (A) were quantified and plotted. 100 cells were counted for each condition. **(C-D)** IF-FISH images and quantification showing POLD3 (green) co-localization with telomeres (red) in the indicated HEK 293T cell lines (n = 100). **(E)** Quantification of relative POLD3 protein levels shown in Figure 7K. n = 3 biological replicates. Data represents the mean ± SD. Statistical significance was calculated using one-way ANOVA with Fisher's LSD tests: $^{****}P<0.0001$.

Figure S9

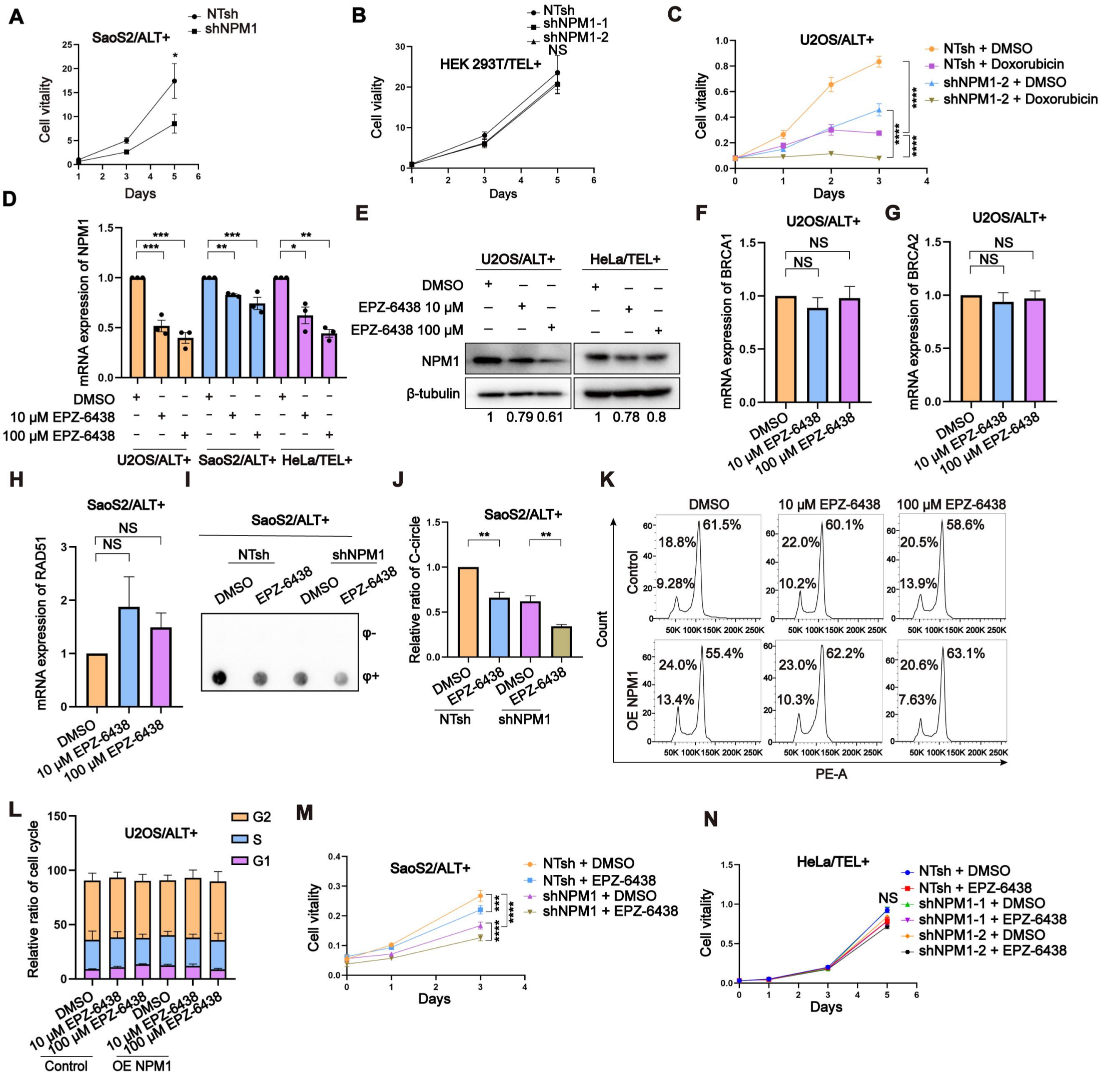

Figure S9. Targeting NPM1 mediated alternative telomere lengthening sensitizes doxorubicin treatment in osteosarcoma.

(A-B) Growth curve determined by CCK-8 assay in SaoS2 and HEK 293T NT shRNA and sh*NPM1* clones for 5 days and the absorbance of OD_{450nm} was detected. **(C)** Growth curve determined by CCK-8 assay. U2OS NT shRNA and sh*NPM1* clones were treated with DMSO or Dox (0.075 μM) for 3 days and the absorbance of OD_{450nm} was detected. **(D)** qRT-PCR showing the mRNA levels of *NPM1* in U2OS, SaoS2 and HeLa cells treated with DMSO or EPZ-6438 (10 μM or 100 μM) for 48 h. **(E)** U2OS and HeLa cells were treated with 10 μM or 100 μM EPZ-6438 for 48 h. Western blotting of NPM1 expression was performed. **(F-H)** qRT-PCR showing the mRNA levels of *BRCA1*, *BRCA2* and *Rad51* in U2OS/SaoS2 cells treated with DMSO or EPZ-6438 (10 μM or 100 μM) for 48 h. **(I-J)** C-circles assay was performed on SaoS2 NT shRNA and sh*NPM1* clones treated with 10 μM EPZ-6438 for 3 days, with 3 biological replicates. The relative levels of C-circles from (I) were quantified. **(K)** Flow cytometry assay showing that U2OS cell cycle arrest in G2/M phase. **(L)** Cell cycle profiles of U2OS control and NPM1 overexpressed cells treated with 10 or 100 μM EPZ-6438 for 48 h. **(M-N)** CCK-8 assay of NT shRNA and sh*NPM1* clones in SaoS2 and HeLa cells treated with DMSO or EPZ-6438 (10 μM)

for 3 or 5 days. Data represents the mean ± SEM. n = 3 biological replicates. $^{*}P<0.05$, $^{**}P<0.01$, $^{***}P<0.001$, $^{****}P<0.0001$. Two tailed t test was used for (A, B, C, M, N and O). One-way ANOVA with Fisher's LSD tests was used for (D F, G H and I).

Table S1

HeLa			U2OS		
Gene	Symbol	Reads	Gene	Symbol	Reads
ORF4941	MGAT2	81305	ORF13750	AURKAIP1	18497
ORF864	TM4SF19	16336	ORF5069	SPHK2	15313
ORF7637	CFP	5808	ORF8189	SNX21	4828
ORF8224	C12orf49	4947	ORF7422	C8orf4	2446
ORF6197	CCND3	4708	ORF5832	FXYD6	1821
ORF91	TSPAN5	3720	ORF5415	LOC158435	1064
ORF71253	JOSD2	3286	ORF1436	WBSCR28	1030
ORF12391	SRPRB	2841	ORF3037	PEX5	826
ORF13218	SUV39H2	2790	ORF3078	NPM1	795
ORF11593	ST3GAL3	2401	ORF6753	UQCRQ	576
ORF8830	RTN1	1979	ORF11593	ST3GAL3	447
ORF3263	TMX2	1891	ORF53112	RAB11B	354
ORF13750	AURKAIP1	1582	ORF9970	AGXT2L2	280
ORF6978	SLC48A1	1465	ORF9194	LDLRAP1	276
ORF5673	PLN	1286	ORF5333	MGC16025	211
ORF4686	TMEM98	1239	ORF5290	RCC1	150
ORF4631	ATP6V0B	1224	ORF56253	C17orf68	135
ORF7568	DHRS13	1198	ORF9447	SLC25A41	128
ORF6065	C12orf57	1138	ORF9510	C6orf218	128
ORF71454	CNIH	1120	ORF7927	PPDPF	127
ORF5802	GNG12	1076	ORF5322	TMEM176A	105
ORF7898	KIAA0368	1044	ORF7219	PCBP3	95
ORF14848	WFDC11	890	ORF13218	SUV39H2	93
ORF6130	SSR3	781	ORF14881	HIST2H2BE	79
ORF593	ROGDI	760	ORF13358		79
ORF7230	TMEM106A	732	ORF6978	SLC48A1	79
ORF4187	ALKBH4	630	ORF10504	ANKRD39	71
ORF108	BCL7B	620	ORF3089	HMGN3	71
ORF235	H2AFY2	602	ORF10931	HS3ST1	71
ORF552	SSSCA1	601	ORF7637	CFP	71
ORF8400	ZNF417	582	ORF5817	DGCR14	71
ORF5732	PPT1	542	ORF3739	SYK	71
ORF3115	DYNLL2	536	ORF5668	CLIC3	71
ORF405	RTN3	530	ORF8268	NCRNA00246B	71
ORF8884	MXD4	498	ORF8031	CALML5	71
ORF9487	C2orf18	482	ORF6318	HIST1H4E	71
ORF3336	RCVRN	477	ORF1896	C2orf40	71
ORF2828	HLA-DQA1	448	ORF6097	LGALS1	64
ORF5450	CD69	445	ORF11472	UPP1	64
ORF10841	PLEKHH2	444	ORF8910	NECAP1	64
ORF2003	SLC6A1	440	ORF3336	RCVRN	64
ORF52774	TAS2R41	435	ORF55747	HIST1H2BF	64
ORF3031	STOM	421	ORF5143	MDFI	64
ORF14381	STX16	406	ORF14898	FAM69A	64
ORF2682	CLDN1	404	ORF1221	OSCP1	60
ORF1082	SLC2A6	381	ORF6044	PDZD11	60
ORF14802	SCD	381	ORF9163	SH2B3	60

ORF56253	C17orf68	199	ORF7568	DHRS13	60
ORF7666	SLC39A14	352	ORF4631	ATP6V0B	60
ORF10931	HS3ST1	346	ORF5543	SAA1	59
ORF8612	F12	346	ORF1082	SLC2A6	59
ORF8565	HEATR2	340	ORF7557	NRG4	59
ORF9194	LDLRAP1	328	ORF183	C9orf80	59
ORF7247	SLC9A1	328	ORF3926	NPDC1	59
ORF1221	OSCP1	323	ORF6513	HMGN2	59
ORF9351	RAB27B	323	ORF7227	SLC7A11	58
ORF10580	NKAIN4	289	ORF14854	SFT2D2	58
ORF4138	PLEKHF1	289	ORF3905	SNRPN	58
ORF15008	TMEM198	287	ORF4994	IGLC1	58
ORF9500	MMGT1	266	ORF55750	HIST3H2BB	58
ORF8223	HIST2H4B	265	ORF393	VSIG4	58
ORF7640	CD48	259	ORF3235	ABLIM1	58
ORF1529	SLC16A7	245	ORF8056	PHPT1	58
ORF14897	CCDC75	244	ORF4113	MYL10	57
ORF3037	PEX5	230	ORF5401	LOC84856	57
ORF13534	HBEGF	223	ORF7492	FTL	57
ORF5318	RBM14	221	ORF54610	MT1DP	57
ORF575	AGA	220	ORF9638	FAM129A	57
ORF11298	DNAI2	211	ORF1021	DIO3	57
ORF6240	PRAF2	206	ORF14072	HIST1H3F	57
ORF7676	GFRA1	203	ORF552	SSSCA1	57
ORF2887	PREB	201	ORF56128	HIST2H2BA	56
ORF636	NTF4	199	ORF679	FAM119B	56
ORF8961	MLLT6	199	ORF4829	KHK	56
ORF522	FAM3D	193	ORF9855	TCP10L	56
ORF9593	MTPN	191	ORF1009	PGLS	56
ORF1387	IZUMO4	178	ORF2005	MOBKL1A	55
ORF1672	COX5A	174	ORF266	RAB38	55
ORF7828	PORCN	167	ORF5624	NDUFS4	54
ORF7846	SPDEF	165	ORF1156	RND2	54
ORF11685	F10	165	ORF575	AGA	53
ORF612	CCDC101	159	ORF7572	LRFN4	53
ORF1828	ASB8	157	ORF9500	MMGT1	52
ORF10213	GLT25D2	157	ORF4852	CRYBA2	52
ORF9422	C1orf190	156	ORF14931	AQP7P2	52
ORF9913	SLC39A11	154	ORF1828	ASB8	50
ORF2680	RNF125	154	IOH11386	PPPDE2	49
ORF294	JOSD1	150	ORF6130	SSR3	49
ORF8184	MTHFS	149	ORF864	TM4SF19	48
ORF5535	COMMD10	147	ORF3014	C14orf126	48
IOH2891	CISD3	145	ORF7057	NDUFA4L2	48
ORF3960	SDF2	145	ORF10841	PLEKHH2	48
IOH56988	TSPAN13	139	ORF11664	VBP1	47
IOH28084	TUBGCP2	138	ORF231	RAN	47
ORF13390	C9orf142	137	ORF2460	RPS29	46
ORF1436	WBSCR28	137	ORF2682	CLDN1	46
ORF6367	KLRG1	128	ORF4138	PLEKHF1	46
ORF8258	RSPO2	126	ORF4686	TMEM98	45

ORF8638	PLCB2	123	ORF54535	H2BFS	45
ORF9598	SFRS17A	121	ORF7640	CD48	45
ORF2408	MRPL42	120	ORF2645	CHIC2	45
ORF4263	PCGF1	119	ORF6430	TNNT1	45
ORF4355	PPPDE1	116	ORF8959		44
ORF71370	ZPLD1	114	IOH57335	ZCCHC3	44
ORF14997	MLLT10	112	ORF12391	SRPRB	44
ORF3199	C3orf75	112	ORF12774	HIST1H3D	44
ORF14872	PIGS	111	ORF4178	ZNF205	44
ORF7953	MMP2	107	ORF53235	SNRPC	44
ORF568	RNF114	105	ORF5490	ZNF273	44
ORF637	WRB	104	ORF56613	LY6E	44
ORF8589	FAM73B	104	ORF6481	HIST1H2BC	43
ORF7876	PTPMT1	102	ORF7138	FZD7	43
ORF11472	UPP1	101	ORF72100	HIST3H3	42
ORF13665	TNFRSF10D	99	ORF14832	AADACL2	42
ORF56613	LY6E	99	ORF4166	NCF1	40
ORF3245	CLDN4	99	ORF4941	MGAT2	39
ORF6467	PDCL	98	ORF1536	DNAJB3	39
ORF7482	SLC35C1	97	ORF3931	C9orf16	38
ORF8495	CYTL1	95	ORF5839	TUBA4A	37
ORF8110	BAT5	95	ORF10213	GLT25D2	37
ORF14931	AQP7P2	93	ORF1237	WBP2NL	35
ORF3838	PAFAH1B3	93	ORF14372	LRRC25	35
ORF9413	IKZF2	93	ORF15044	LYSMD1	35
ORF14501	C22orf40	92	ORF1537	SCRG1	34
ORF6513	HMGN2	88	ORF1556	GIMAP7	33
ORF71442	IRF2BP1	88	ORF2002	KIAA2013	32
ORF2556	HLA-DMB	87	ORF2080	FANCM	32
ORF2317	VAT1L	83	ORF235	H2AFY2	32
ORF7138	FZD7	83	ORF2434	NCOA4	32
ORF14305	PRDX2	82	ORF2556	HLA-DMB	32
ORF6668	HLA-DRB5	81	ORF2670	MR1	32
ORF1556	GIMAP7	80	ORF2845	REEP6	30
ORF6916	TNFRSF1A	79	ORF3463	MRPL34	30
ORF4997	EXOSC5	78	ORF3960	SDF2	30
ORF5226	PCNXL2	76	ORF4173	C1orf35	29
ORF7951	DDOST	76	ORF4765	ITGB2	29
ORF8211	THAP6	76	ORF4940	SLC12A7	29
ORF14881	HIST2H2BE	75	ORF4956	PSEN2	29
ORF14993	ESPNL	75	ORF52774	TAS2R41	28
ORF4325	S100A14	74	ORF53047	SRRT	28
ORF5258	ZNF678	73	ORF53258	SMARCA4	28
ORF3396	NME3	73	ORF54388	PI15	28
ORF1191	FABP6	71	ORF5450	CD69	27
ORF1589	ADAM30	71	ORF54767	SPANXN4	27
ORF14784	ANKRD12	70	ORF55203	FN1	27
ORF4316	GJB5	70	ORF55456		27
ORF10143	SVOPL	70	ORF5550	FXYD2	27
ORF2818	C1QC	70	ORF5751	HLA-DRB1	26
ORF14894	CD40LG	68	ORF6014	ASS1	26

ORF6123	EXOSC7	68	ORF6274	FXC1	26
ORF4728	RNPS1	68	ORF636	NTF4	26
ORF5654	REG1A	68	ORF6755	CIAO1	26
ORF6290	NAT8	68	ORF70418	HIST1H3I	25
ORF2845	REEP6	67	ORF71381	SLCO1A2	25
ORF9204	SIRPD	66	ORF7220	MRPS24	25
ORF3695	HIST2H2AA3	65	ORF7553	C14orf147	25
ORF11968	NCR1	65	ORF7920	SILV	25
ORF14758	NUDT14	65	ORF8110	BAT5	24
ORF6288	DCPS	64	ORF8144	C9orf102	24
ORF5069	SPHK2	63	ORF8395	CD1D	24
ORF1098	CXCL6	63	ORF8589	FAM73B	24
ORF9364	ABCC11	63	ORF8968	NF2	24
ORF307	RBBP9	62	ORF9089	TTLL3	24
ORF2037	NME5	62	ORF9204	SIRPD	24
ORF5617	TSPAN8	62	ORF9364	ABCC11	24
ORF5984	SC5DL	62	ORF9913	SLC39A11	24
ORF10711	FBXO4	61	IOH46484	OR51I1	24
ORF3347	MEA1	61	ORF13665	TNFRSF10D	23
ORF71558	FUT1	61	ORF14947	FTHL3	23
ORF2670	MR1	60	ORF1672	COX5A	23
ORF4961	SH3BGR	60	ORF3169	PSMD4	23
ORF7920	SILV	59	ORF3304	STAG2	23
ORF548	ZDHHC3	58	ORF4464	EIF5A	23
ORF2815	HSD17B8	58	ORF5074	TINF2	23
ORF5750	GINS4	57	ORF544	RPS14	23
IOH23024	FITM2	57	ORF5802	GNG12	22
ORF5543	SAA1	56	ORF71452	LEPRE1	22
ORF116	RAB39B	56	ORF7501	HAX1	22
ORF8055	C22orf23	56	ORF7609	RHOT2	22
ORF8227	TMEM86B	56	ORF2463	CBFA2T2	22
ORF9855	TCP10L	56	ORF2607	TMEM148	22
ORF5195	GPR108	55	ORF2721	RHOD	22
ORF14854	SFT2D2	52	ORF4090	ATG5	22
ORF545	RPS5	52	ORF5643	METTL2A	22
ORF384	LOH12CR1	51	ORF5664	LSM3	22
ORF6301	ZNF215	50	ORF56664	SPRY4	22
ORF657	S100A3	50	ORF8476	PRR15	22
ORF3715	UBE2E3	48	ORF6097	LGALS1	22
ORF6430	TNNT1	48	ORF11620	PSMA2	22
ORF8634	PAPOLA	48	ORF5401	LOC84856	22
ORF7190	HLA-DQB1	47	ORF8386	FGFBP2	21
ORF7553	C14orf147	47	IOH10046	FLYWCH1	21
ORF1033	CCDC134	46	IOH57135	PEX1	21
ORF2911	NAT15	46	ORF1004	ARL8A	21
ORF4765	ITGB2	46	ORF10061	KHDRBS3	21
ORF8017	SLC39A7	45	ORF12358	CDKN3	21
ORF8486	CYGB	44	ORF12995	LHFP	21
ORF7997	S100A13	44	ORF13436	C19orf40	21
ORF1067	ZNF580	44	ORF14266	ABCA9	21
ORF4362	C9orf89	44	ORF14633	RAB1B	21

ORF4162	C11orf10	43	ORF1648	KHDC1	21
ORF7266	C19orf60	43	ORF186	UBR7	21
ORF744	DCAKD	43	ORF2129	RBM12B	21
ORF8490	TMEM61	43	ORF234	ZNF707	20
ORF70425	CYP1A2	42	ORF2585	RPS13	20
ORF70621	C2orf66	42	ORF2627	GNS	20
ORF5392	C9orf70	41	ORF2907	NTRK3	20
ORF10811	LYPD5	41	ORF2924	OLFM2	20
ORF3851	ARF5	40	ORF2962	C14orf79	20
ORF341	C18orf18	40	ORF3114	CST7	20
ORF3027	SLC19A2	39	ORF3584	C12orf44	20
ORF2032	RHOXF2	38	ORF401	NARF	20
ORF2486	PIK3R3	38	ORF420	CLU	20
ORF3535	EMD	37	ORF4212	CDK2AP2	20
ORF1617	C7orf45	37	ORF443	CENPH	19
ORF6125	RILPL2	36	ORF5179	B3GAT3	19
ORF3686	SIRT2	36	ORF5255	PLAT	19
ORF4611	C17orf62	36	ORF5290	RCC1	19
ORF266	RAB38	35	ORF54034	SEC31A	19
ORF10216	MBLAC2	35	ORF5487	ANAPC13	19
ORF5322	TMEM176A	35	ORF5505	CPA2	19
ORF697	TESC	35	ORF5527	DCN	19
ORF9035	GNA11	35	ORF55677	SYT15	19
ORF587	EGFL7	34	ORF5578	TFPI2	19
ORF71254	TMEM81	34	ORF55954	PEX11A	19
ORF8148	PACRGL	34	ORF56125	HSDL1	18
ORF2803	HMBS	33	ORF56377	LOC284749	18
ORF5550	FXYD2	33	ORF56468	C1orf161	18
ORF4324	TMEM39B	33	ORF6382	MED9	18
ORF5407	TMEM41A	33	ORF647	BAT2L1	18
ORF6645	DHRS12	33	ORF665	PSMD10	18
ORF6922	RTN4R	33	ORF6808	FKBP8	18
ORF71650	FAM92B	33	ORF70571	ALG10B	18
ORF2861	C9orf46	32	ORF71098	FAM19A5	18
ORF3059	IL1B	32	ORF71999	CD28	18
IOH12316	OTUD7B	31	ORF7277	SLC39A8	18
ORF2416	EBP	31	ORF8279	LECT1	18
ORF2663	UBD	31	ORF8959		18
ORF3800	EPHX1	31	ORF8982	ZNF3	18
ORF5973	MANBAL	31	ORF9020	PDE4D	17
ORF8245	RDH12	31	ORF9290	CCDC42	17
ORF8395	CD1D	31	ORF9324	FMO3	17
ORF5624	NDUFS4	30	ORF946	DEGS1	17
ORF2113	ACSS1	30	ORF9745	SPIC	17
ORF4624	TSPAN15	30	ORF12038	HIST2H2AC	17
ORF5935	RAC2	30	ORF55747	HIST1H2BF	17
ORF7422	C8orf4	29	ORF55750	HIST3H2BB	17
ORF5736	C1orf97	29	ORF5667	ANKRD36BP1	17
ORF2812	DOK5	29	ORF6807	NFKBIE	17
ORF9211	FAM131C	29	ORF71955	C20orf79	17
ORF11516	COMTD1	28	ORF9866	ZMYND10	17

ORF2053	TMEM217	28	ORF9970	AGXT2L2	17
ORF3079	IL24	28	ORF13167	KCTD6	17
ORF4284	NDFIP1	28	ORF6994	SLC35B1	17
ORF5311	PYCRL	28	ORF8644	FAM175A	17
ORF7127	HOMER3	28	ORF2411	TCEAL4	16
ORF4829	KHK	27	ORF3593	PRDX4	16
ORF3063	AQP3	27	IOH22243	PEX12	16
ORF13514	WNT7B	27	IOH28234	RAB24	16
ORF8058	POLDIP3	27	IOH28510	ATN1	16
ORF9527	C3orf24	27	IOH39839	PCDHB12	16
ORF9862	SPZ1	27	IOH50149	STYXL1	16
ORF6318	HIST1H4E	26	IOH59717	SGIP1	16
ORF11182	COX7B2	26	IOH7446	TBX21	16
ORF1424	GNPTG	26	IOH9913	BECN1	16
ORF2570	C16orf79	26	ORF10296	C1orf74	16
ORF3904	MALL	26	ORF11160	APOBEC3F	16
ORF7572	LRFN4	26	ORF11300	ITM2A	16
ORF3729	MRPL11	25	ORF11550	RNF144A	16
ORF6358	S100A4	25	ORF13293	FUT6	16
ORF13081	SERPINC1	25	ORF13310	UGT1A9	16
ORF1381	C6orf64	25	ORF14053	GNRH2	16
ORF5713	ROMO1	25	ORF14596	GPR61	16
ORF70936	PPP1R2P9	24	ORF14692	TMBIM4	16
ORF3655	NRBP1	24	ORF14932	IFNG	16
ORF3112	GSTM4	24	ORF1737	C8orf68	16
ORF6270	TTYH1	24	ORF1928	C10orf46	16
ORF11922	MYOG	23	ORF2068	GRID1	16
ORF1736	ANKRD29	23	ORF2127	HLF	16
ORF2677	CA6	23	ORF2328	FMR1NB	16
ORF287	BCL2A1	23	ORF2833	SPATA7	15
ORF52603	NCRNA00247	23	ORF2865	SFRP2	15
ORF9514	GAL	23	ORF2922	IGFBP5	15
ORF53112	RAB11B	22	ORF297	RAB18	15
ORF3140	EI24	22	ORF3046	GALNT14	15
ORF134	CCL2	22	ORF3087	CAV1	15
ORF1820	MGMT	22	ORF3289	GOLM1	15
ORF2257	NUDT16	22	ORF3346	HIST3H2A	15
ORF2412	ST3GAL4	22	ORF363	HNRNPA0	15
ORF2813	CCL5	22	ORF3839	TMEM176B	15
ORF3473	SPIN2B	22	ORF3961	MIF	15
ORF3832	MRTO4	22	ORF415	LOC541471	15
ORF9778	FAM119A	22	ORF416	TSPYL4	15
ORF6972	MRPS6	21	ORF4165	CMTM6	15
ORF10860	FAM3B	21	ORF4315	CYB5B	15
ORF4554	TRAPPC3	21	ORF4379	SUSD4	15
ORF11028	SRSF2	21	ORF4436	TSPAN14	15
ORF3334	INPP5K	21	ORF4707	MAD2L1	15
ORF6389	C6orf203	21	ORF5139	TMEM141	15
ORF71970	TAS2R19	21	ORF5213	ABHD14B	15
ORF7797	ERAL1	21	ORF52642	C21orf84	15
ORF7509	SH3BGRL	20	ORF5333	MGC16025	15

IOH13446	BEAN	20	ORF5364	DCUN1D5	15
ORF2637	HLA-DQB2	20	ORF5457	SH3YL1	15
ORF824	MEOX2	20	ORF5484	RPA3	15
ORF9574	BEST3	20	ORF55281	RBMS1	15
ORF9965	EFNA1	20	ORF5740	EVI2B	15
ORF407	S100A16	19	ORF5781	PSG4	15
ORF4003	AGPAT1	19	ORF5885	IFI6	15
ORF56557	HLA-DQA1	19	ORF616	ITGB1BP1	14
ORF8666	F8	19	ORF6257	TMEM45B	14
ORF8355	GSDMB	18	ORF6275	CLEC3B	14
ORF3256	NUP85	18	ORF6483	UQCRC1	14
ORF3557	ATP1B1	18	ORF6773	TM4SF4	14
ORF55512	C17orf74	18	ORF6858	SAT2	14
ORF6450	ALX1	18	ORF70540	OR13G1	14
ORF7273	LAT	18	ORF70632	OR51Q1	14
ORF8255	TRAV20	18	ORF71584	PRPH2	14
ORF6012	PSMB3	17	ORF71817	COX18	14
ORF3078	NPM1	17	ORF7325	CTHRC1	14
ORF15044	LYSMD1	17	ORF7408	TM4SF18	14
ORF2301	DDX51	17	ORF7473	GSTZ1	14
ORF3359	NSDHL	17	ORF7629	NKG7	14
ORF645	PEX11B	17	ORF7687	SERPINA4	14
ORF8186	CLEC2D	17	ORF8419	CCRL2	14
ORF8303	ITM2C	17	ORF8421	PNLIPRP1	14
ORF1590	RPL37A	16	ORF8908	IP6K2	14
ORF6184	ADPRHL1	16	ORF8960	RXRA	14
ORF9760	C9orf100	16	ORF9021	PMS1	14
ORF1557	HSPA13	16	ORF9072	GNG10	14
ORF3918	VTI1B	16	ORF9123	RMND1	13
ORF53377	C19orf75	16	ORF9131	PKD1L2	13
ORF54599	UTS2	16	ORF9199	PNOC	13
ORF71091	VSIG1	16	ORF9429	HSD3B2	13
ORF7718	PROCR	16	ORF954	YIF1A	13
ORF855	MRPS2	16	ORF9652	KLHL36	13
ORF5490	ZNF273	15	ORF9849	GABRD	13
ORF10179	ICK	15	IOH50202	GLA	13
ORF10491	CMTM8	15	IOH57335	ZCCHC3	13
ORF12247	TMEM53	15	ORF10110	RGL2	13
ORF14684	ARFGAP1	15	ORF10862	ST6GALNAC3	13
ORF1537	SCRG1	15	ORF113	EEF1A1	13
ORF2012	LHFPL5	15	ORF14273	SPRY1	13
ORF3246	TRNAU1AP	15	ORF14922	RPS17	13
ORF4306	LIMD2	15	ORF1540	LZTFL1	13
ORF52902	KIR3DL2	15	ORF1618	IFIT2	13
ORF760	C18orf10	15	ORF232	H3F3B	13
ORF7821	NAT14	15	ORF2573	PTPRA	13
ORF9526	MFSD7	15	ORF3304	STAG2	13
ORF9895	MIF4GD	15	ORF5074	TINF2	13
ORF14830	PFDN5	14	ORF5160	STARD7	13
ORF236	UBE2C	14	ORF5164	RPS11	13
ORF2860	PRTFDC1	14	ORF6215	LEPREL2	13

ORF5406	DYDC2	14	ORF644	ITPK1	13
ORF5486	PRDX1	14	ORF6647	NUP160	13
ORF55995	TAS2R14	14	ORF6908	STX10	13
ORF715	TWSG1	14	ORF7010	PFDN1	13
ORF721	UCHL3	14	ORF7559	MRPS36	12
ORF7632	TPST2	14	ORF771	HDGFRP3	12
ORF890	H1F0	14	ORF9004	CHP	12
ORF8345	IL2RB	13	ORF2404	ATP5B	12
ORF3628	ESD	13	ORF2460	RPS29	12
ORF6315	LYRM2	13	ORF3137	MLEC	12
IOH13343	CARD17	13	ORF38	RPLP1	12
ORF13389	C15orf57	13	ORF5559	EIF1B	12
ORF14110	ORMDL3	13	ORF5559	LOC388387	12
ORF2843	LYPLA1	13	ORF9163	SH2B3	12
ORF3953	FADS3	13	IOH4003	GRIK3	12
ORF4738	COX4NB	13	IOH42047	FEM1A	12
ORF55517	C4orf32	13	ORF10180	ST3GAL2	12
ORF5960	PRCP	13	ORF102	CLDN11	12
ORF7558	NPRL2	13	ORF10511	NSUN7	12
ORF1156	RND2	12	ORF10529	ASB17	12
ORF8056	PHPT1	12	ORF10733	INO80C	12
IOH44740	POU1F1	12	ORF11058	CNST	12
ORF10751	DHDPSL	12	ORF11289	CRCP	12
ORF331		12	ORF1151	CD200	12
ORF501	RBM8A	12	ORF11522	ANKS3	12
ORF5501	HLA-DRB4	12	ORF11643	RPS6KA4	12
ORF6035	SCARB1	12	ORF11958	DHRS9	11
ORF6837		12	ORF12088	FAM173B	11
ORF6853	MBD4	12	ORF122	STX8	11
ORF7091	SNAPC2	12	ORF12382	SPATA20	11
ORF8006	DERL1	12	ORF12623	KDELR1	11
ORF2135	CDRT4	11	ORF1276	TSPAN7	11
ORF14927	CCR4	11	ORF12963	ALG2	11
ORF10656	TMEM17	11			
ORF2814	C9orf30	11			
ORF3856	UCHL1	11			
ORF4591	CLTB	11			
ORF53212	ROPN1	11			
ORF5741	MIA	11			
ORF6870	CHMP6	11			
ORF72087	GNG8	11			
ORF8393	PPY	11			
ORF8401	GP9	11			
ORF8512	GOSR2	11			
ORF9680	FAM190B	11			
ORF9904	GPX7	11			
ORF14072	HIST1H3F	10			
ORF8838	OTUB2	10			
ORF6753	UQCRQ	10			
IOH44600	PHGDH	10			
ORF1207	DPPA2	10			

ORF12362	REEP5	10			
ORF2656	ZMYND19	10			
ORF2807	HLA-DRB3	10			
ORF3248	C1orf56	10			
ORF3370	DBN1	10			
ORF5823	GRB7	10			
ORF5899	IMPA2	10			
ORF6255	TMED7	10			
ORF6755	CIAO1	10			
ORF71904	IL12A	10			
ORF7236	LMF2	10			
ORF7384		10			
ORF8729	CCNL1	10			
ORF9929	CCNG2	10			

Table S2

Description	Sum PEP Score	Coverage [%]	# Peptides	# PSMs	# Unique Peptides	# AAs	MW [kDa]	calc. pI	Score Sequest HT: Sequest HT
60S ribosomal protein L3=RPL3	110.584	48	29	77	29	403	46.1	10.2	144
Lupus La protein OS=SSB	23.644	22	10	16	10	408	46.8	7.12	15.13
Heterogeneous nuclear ribonucleoprotein F=HNRNPF	23.996	17	5	11	4	415	45.6	5.58	14.41
Putative RNA-binding protein Luc7-like 2=LUC7L2	25.049	24	9	16	5	392	46.5	10	13.83
rRNA methyltransferase 3, mitochondrial=MRM3	15.403	20	9	10	9	420	47	8.73	12.17
Putative RNA-binding protein Luc7-like 1=LUC7L	11.628	17	5	7	1	371	43.7	9.92	8.47
Serpin B12=SERPINB12	9.862	14	5	7	5	405	46.2	5.53	5.69
Splicing factor 3B subunit 4=SF3B4	2.109	3	1	2	1	424	44.4	8.56	4.58
Interleukin enhancer-binding factor 2=ILF2	5.799	10	3	6	3	390	43	5.26	4.11
Alpha-enolase OS=ENO1	3.784	6	2	2	2	434	47.1	7.39	3.39
Polymerase delta-interacting protein 3=POLDIP3	5.457	11	3	3	3	421	46.1	9.99	2.87
p21-activated protein kinase-interacting protein 1=PAK1IP1	8.609	13	5	5	5	392	43.9	8.91	2.35
Putative methyltransferase C9orf114=SPOUT1	2.442	3	1	2	1	376	42	7.43	2.32
Smad nuclear-interacting protein 1=SNIP1	5.507	7	3	4	3	396	45.8	9.99	2.06
WD repeat-containing protein 55=WDR55	1.515	3	1	1	1	383	42	4.92	1.98
KRR1 small subunit processome component homolog=KRR1	3.051	6	2	2	2	381	43.6	9.77	1.92
DnaJ homolog subfamily A member 2=DNAJA2	1.175	2	1	1	1	412	45.7	6.48	1.9
Eukaryotic initiation factor 4A-III=EIF4A3	2.887	6	2	2	2	411	46.8	6.73	1.9
Cathepsin D=CTSD	3.321	4	2	2	2	412	44.5	6.54	1.89
DnaJ homolog subfamily A member 1=DNAJA1	1.309	3	1	2	1	397	44.8	7.08	1.65
Splicing factor 45=RBM17	2.99	6	3	3	3	401	44.9	5.97	1.62
Cell growth-regulating nucleolar protein=LYAR	8.631	14	4	5	4	379	43.6	9.57	0
Eukaryotic initiation factor 4A-II=EIF4A2	2.242	3	1	1	1	407	46.4	5.48	0
DNA-directed RNA polymerase I subunit RPA49=POLR1E	1.172	2	1	1	1	419	47.2	8.94	0
WD repeat-containing protein 18=WDR18	4.003	6	2	2	2	432	47.4	6.7	0

Table S3

Drug name	Concentration	mRNA expression of NPM1
Tazemetostat	50 µM	15.90%
Bobcat339	50 µM	43%
Phthalimido-L-tryptophan	50 µM	57.40%
UNC0631	50 µM	59.80%
Birabresib	50 µM	63.20%
SP2509	50 µM	67.20%
3-TYP	50 µM	70%
Sodium butanoate	50 µM	72.50%
AZD5153 6-Hydroxy-2-naphthoic acid	50 µM	73.20%
MS436	50 µM	77%
SGC-CBP30	50 µM	81.30%
UNC1999	50 µM	85.60%
Citarinostat	50 µM	88.30%
GSK 5959	50 µM	89.20%
